# Trans-ethnic Mendelian-randomization study reveals causal relationships between cardiometabolic factors and chronic kidney disease

**DOI:** 10.1093/ije/dyab203

**Published:** 2021-10-20

**Authors:** Jie Zheng, Yuemiao Zhang, Humaira Rasheed, Venexia Walker, Yuka Sugawara, Jiachen Li, Yue Leng, Benjamin Elsworth, Robyn E Wootton, Si Fang, Qian Yang, Stephen Burgess, Philip C Haycock, Maria Carolina Borges, Yoonsu Cho, Rebecca Carnegie, Amy Howell, Jamie Robinson, Laurent F Thomas, Ben Michael Brumpton, Kristian Hveem, Stein Hallan, Nora Franceschini, Andrew P Morris, Anna Köttgen, Cristian Pattaro, Matthias Wuttke, Masayuki Yamamoto, Naoki Kashihara, Masato Akiyama, Masahiro Kanai, Koichi Matsuda, Yoichiro Kamatani, Yukinori Okada, Robin Walters, Iona Y Millwood, Zhengming Chen, George Davey Smith, Sean Barbour, Canqing Yu, Bjørn Olav Åsvold, Hong Zhang, Tom R Gaunt

**Affiliations:** 1 MRC Integrative Epidemiology Unit (IEU), Bristol Medical School, University of Bristol, Oakfield House, Oakfield Grove, Bristol, UK; 2 Renal Division, Peking University First Hospital, Peking University Institute of Nephrology, Key Laboratory of Renal Disease, Ministry of Health of China, Key Laboratory of Chronic Kidney Disease Prevention and Treatment (Peking University), Ministry of Education, Beijing, P. R. China; 3 K.G. Jebsen Center for Genetic Epidemiology, Department of Public Health and Nursing, NTNU, Norwegian University of Science and Technology, Trondheim, Norway; 4 Department of Surgery, University of Pennsylvania Perelman School of Medicine, Philadelphia, PA, USA; 5 Division of Nephrology and Endocrinology, The University of Tokyo Hospital, Tokyo, Japan; 6 Department of Epidemiology and Biostatistics, School of Public Health, Peking University, Beijing, P. R. China; 7 Department of Psychiatry, University of California, San Francisco, CA, USA; 8 MRC Biostatistics Unit, Cambridge Institute of Public Health, Cambridge, UK; 9 Cardiovascular Epidemiology Unit, Department of Public Health and Primary Care, University of Cambridge, Cambridge, UK; 10 Department of Clinical and Molecular Medicine, NTNU, Norwegian University of Science and Technology, Trondheim, Norway; 11 Department of Thoracic Medicine, St. Olavs Hospital, Trondheim University Hospital, Trondheim, Norway; 12 Department of Nephrology, St. Olavs Hospital, Trondheim University Hospital, Trondheim, Norway; 13 Department of Epidemiology, University of North Carolina, Chapel Hill, NC, USA; 14 Division of Musculoskeletal and Dermatological Sciences, University of Manchester, Manchester, UK; 15 Institute of Genetic Epidemiology, Department of Biometry, Epidemiology and Medical Bioinformatics, Faculty of Medicine and Medical Center–University of Freiburg, Freiburg, Germany; 16 Eurac Research, Institute for Biomedicine (affiliated with the University of Lübeck), Bolzano, Italy; 17 Tohoku Medical Megabank Organization and Tohoku University Graduate School of Medicine, Tohoku University, Sendai, Miyagi, Japan; 18 Department of Nephrology and Hypertension, Kawasaki Medical School, Kurashiki, Okayama, Japan; 19 Laboratory for Statistical Analysis, RIKEN Center for Integrative Medical Sciences, Yokohama, Japan; 20 Department of Ophthalmology, Graduate School of Medical Sciences, Kyushu University, Fukuoka, Japan; 21 Department of Biomedical Informatics, Harvard Medical School, Boston, MA, USA; 22 Department of Statistical Genetics, Osaka University Graduate School of Medicine, Suita, Japan; 23 Department of Computational Biology and Medical Sciences, Graduate school of Frontier Sciences, the University of Tokyo, Tokyo, Japan; 24 Laboratory of Complex Trait Genomics, Department of Computational Biology and Medical Sciences, Graduate School of Frontier Sciences, the University of Tokyo, Tokyo, Japan; 25 Laboratory of Statistical Immunology, Immunology Frontier Research Center (WPI-IFReC), Osaka University, Suita, Japan; 26 Integrated Frontier Research for Medical Science Division, Institute for Open and Transdisciplinary Research Initiatives, Osaka University, Suita, Japan; 27 Clinical Trial Service Unit and Epidemiological Studies Unit, Nuffield Department of Population Health, University of Oxford, Oxford, UK; 28 NIHR Biomedical Research Centre at the University Hospitals Bristol NHS Foundation Trust and the University of Bristol, UK; 29 Division of Nephrology, University of British Columbia, Vancouver, British Columbia, Canada; 30 British Columbia Provincial Renal Agency, Vancouver, British Columbia, Canada; 31 Department of Endocrinology, Clinic of Medicine, St. Olavs Hospital, Trondheim University Hospital, Trondheim, Norway

**Keywords:** chronic kidney disease, cardiometabolic risk factors, Mendelian randomization, causality, trans-ethnic study

## Abstract

**Background:**

This study was to systematically test whether previously reported risk factors for chronic kidney disease (CKD) are causally related to CKD in European and East Asian ancestries using Mendelian randomization.

**Methods:**

A total of 45 risk factors with genetic data in European ancestry and 17 risk factors in East Asian participants were identified as exposures from PubMed. We defined the CKD by clinical diagnosis or by estimated glomerular filtration rate of <60 ml/min/1.73 m^2^. Ultimately, 51 672 CKD cases and 958 102 controls of European ancestry from CKDGen, UK Biobank and HUNT, and 13 093 CKD cases and 238 118 controls of East Asian ancestry from Biobank Japan, China Kadoorie Biobank and Japan-Kidney-Biobank/ToMMo were included.

**Results:**

Eight risk factors showed reliable evidence of causal effects on CKD in Europeans, including genetically predicted body mass index (BMI), hypertension, systolic blood pressure, high-density lipoprotein cholesterol, apolipoprotein A-I, lipoprotein(a), type 2 diabetes (T2D) and nephrolithiasis. In East Asians, BMI, T2D and nephrolithiasis showed evidence of causality on CKD. In two independent replication analyses, we observed that increased hypertension risk showed reliable evidence of a causal effect on increasing CKD risk in Europeans but in contrast showed a null effect in East Asians. Although liability to T2D showed consistent effects on CKD, the effects of glycaemic phenotypes on CKD were weak. Non-linear Mendelian randomization indicated a threshold relationship between genetically predicted BMI and CKD, with increased risk at BMI of >25 kg/m^2^.

**Conclusions:**

Eight cardiometabolic risk factors showed causal effects on CKD in Europeans and three of them showed causality in East Asians, providing insights into the design of future interventions to reduce the burden of CKD.

Key Messages

- This large-scale genetic study found robust evidence to support the causal roles of eight cardiometabolic risk factors on chronic kidney disease (CKD) among Europeans and three of these risk factors were causal among East Asians.
- Trans-ethnic comparison suggested that hypertension showed a strong causal role on CKD in Europeans but no substantial role in East Asians.
- The genetic evidence suggested that type 2 diabetes may have glucose-independent mechanisms to influence CKD.
- This study highlighted importance of controlling the multimorbidity of cardiovascular disease and CKD as an intervention strategy to reduce the burden of both diseases.

## Introduction

Chronic kidney disease (CKD) affects 10–15% of the population worldwide. It has a major effect on global health, both as a direct cause of morbidity and mortality, and as an important complication for cardiometabolic diseases.^1–3^ From 1990 to 2017, the global age-standardized mortality for many important non-communicable diseases has declined. For example, cardiovascular disease mortality has been reduced by 30.4%. However, the mortality decline for CKD has been just 2.8%.^4^ The majority of interventional trials have focused on disease treatment rather than primary prevention. In the literature, impaired fasting glucose, high blood pressure and high body mass index (BMI) are among the leading risk factors for CKD. However, even with existing interventions for these risk factors, the burden of CKD has not declined as expected.^4^ Moreover, CKD awareness is limited among the general public and healthcare authorities. Thus, a systematic assessment of the causal determinants of CKD is urgently needed to promote a shift from the treatment of CKD patients to the prevention of the disease in high-risk groups.

Well-designed randomized–controlled trials (RCTs) are usually the best approach to estimate a causal relationship between a risk factor and a disease. Whereas several studies have identified risk factors for CKD progression, there is a lack of reliable evidence to support their causal roles on CKD incidence. Mendelian randomization (MR) is an epidemiological method that can be used to obtain evidence about the causal effects of modifying intervention targets.^5^ MR exploits the random allocation of genetic variants at conception and is therefore less susceptible to confounding and reverse causality than traditional observational studies. The increasing availability of genetic-association resources provides a timely opportunity to test the causal effects of various risk factors on CKD.^6^^,^^7^

In this study, we aimed to investigate the causal effects of 45 previously reported risk factors on CKD using two-sample linear and non-linear MR approaches. We used the largest available genome-wide association studies (GWASs) for risk factors in European and East Asian ancestries. Summary data for CKD and estimated glomerular filtration rate (eGFR) were obtained from >1 million participants from the CKDGen consortium,^8^ UK Biobank,^9^ Trøndelag Health (HUNT) Study,^10^ Biobank Japan,^11^ China Kadoorie Biobank^12^ and Japan-Kidney-Biobank/ToMMo consortium.

## Methods

The data, analytic methods and study materials will be made available to other researchers for purposes of reproducing the results. For more details, the genetic-association data of the selected risk factors are available in Supplementary Tables (available as Supplementary data at *IJE* online). The GWAS summary statistics for CKD and eGFR that were generated using UK Biobank and CKDGen data are available from the MRC-IEU OpenGWAS database (https://gwas.mrcieu.ac.uk/) and CKDGen website (http://ckdgen.imbi.uni-freiburg.de/) respectively. The GWAS results from HUNT, Biobank Japan, China Kadoorie Biobank and Japan-Kidney-Biobank/ToMMo can be accessed by request to the data holders. The analytical script of the MR analysis conducted in this study is available via the GitHub repository of the ‘TwoSampleMR’ R package (https://github.com/MRCIEU/TwoSampleMR/).

### Study design

**Figure 1:**
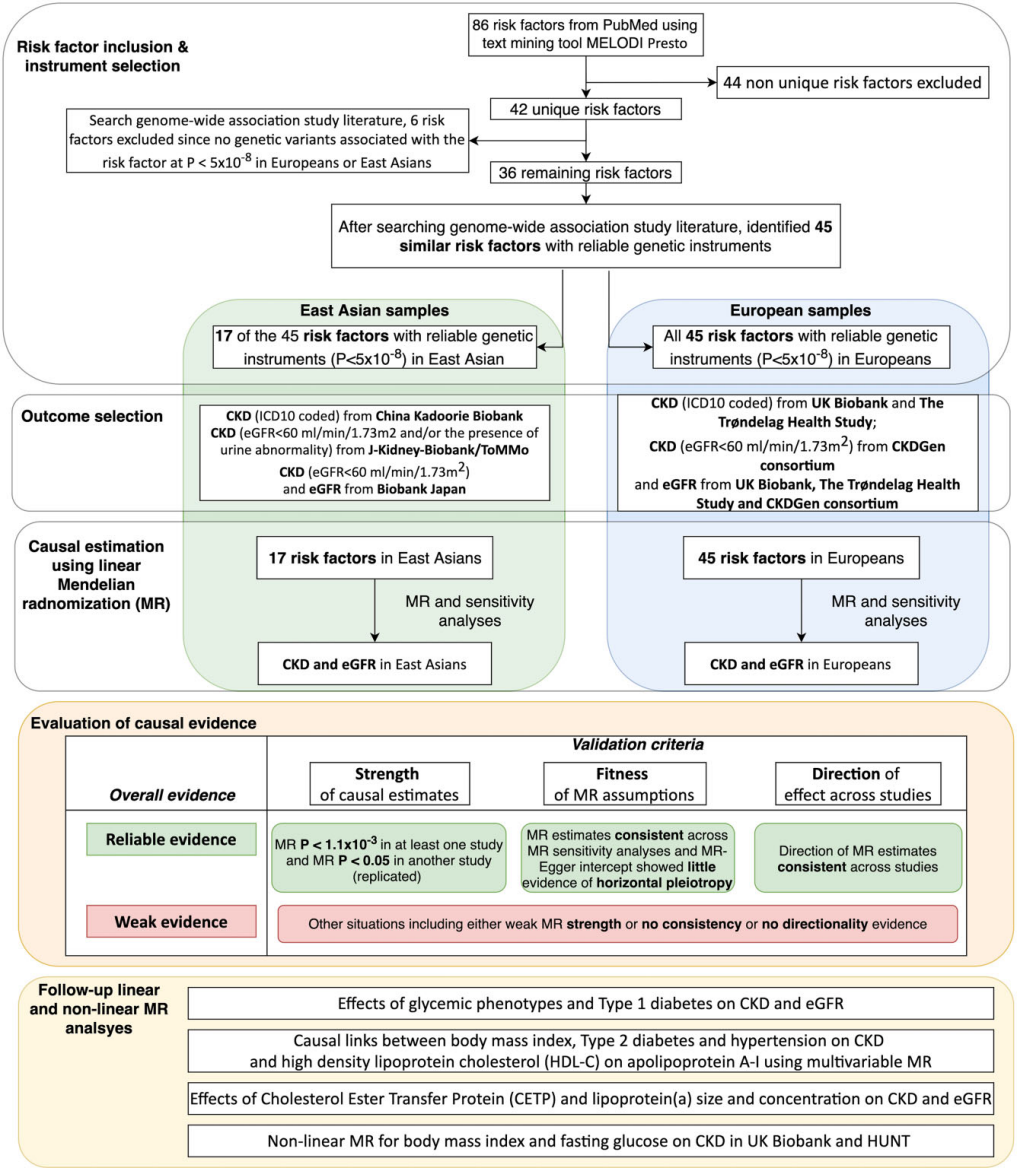
Study design of the trans-ethnic Mendelian-randomization study of chronic kidney disease CKD, chronic kidney disease; eGFR, estimated glomerular filtration rate.

Our study consisted of four components (Figure  1). First, we identified 45 risk factors for CKD by mining PubMed. Second, we estimated the causal effects of these risk factors on CKD and eGFR in CKDGen,^8^ UK Biobank,^9^ HUNT Study,^10^ Biobank Japan,^11^ China Kadoorie Biobank^12^ and Japan-Kidney-Biobank/ToMMo consortium separately. Third, we evaluated the findings based on the strength and consistency of the evidence across MR methods and across individual studies. Finally, we conducted extensive follow-up analyses to confirm the findings for blood pressure, glycaemic and blood lipid phenotypes on CKD. Finally, non-linear MR was performed to estimate the optimal BMI and fasting glucose levels for reducing CKD risk in UK Biobank and the HUNT Study.

### Selection of risk factors

CKD risk factors were identified from a literature review using MELODI-Presto^13^^,^^14^ to search the PubMed database (Supplementary Note S1, available as Supplementary data at *IJE* online). We identified 45 risk factors for CKD, including blood-pressure phenotypes, glycaemic phenotypes, lipid phenotypes, obesity, smoking, alcohol intake, sleep disorders, nephrolithiasis, serum uric acid, coronary artery disease, bone mineral density, homocysteine, C-reactive protein, micro-nutrient phenotypes (serum metals and vitamins), dehydration and thyroid phenotypes. By searching the largest available GWASs (ensuring minimum sample overlap with the outcome samples), we extracted genetic variants associated with all 45 risk factors from European ancestry studies and extracted 17 of the 45 risk factors from East Asian ancestry studies (Supplementary Table S1 and Supplementary Note S1, available as Supplementary data at *IJE* online). To select the independent genetic variants, the genome-wide significant single-nucleotide polymorphism (SNPs) were grouped by linkage disequilibrium (LD) (*r*^2^ <0.001 for SNPs within 1 Mb genomic region) and the SNP with the lowest *P*-value per group was retained (Supplementary Tables S2 and S3, available as Supplementary data at *IJE* online).

### Association of genetic variants with CKD and eGFR

In UK Biobank,^9^ HUNT Study^10^ and China Kadoorie Biobank,^12^ CKD was defined according to the International Classification of Diseases (ICD) 10th Revision. The CKD cases were defined as participants with ICD 10 code N18. The participants with any type of kidney conditions (N00 to N29) were excluded from the controls to reduce the possibility of including CKD cases in the control group. In Japan-Kidney-Biobank/ToMMo consortium, CKD was defined as eGFR of <60 ml/min/1.73 m^2^ and/or the presence of urine abnormality, which is similar to the clinical diagnosis for CKD. In CKDGen^8^ and Biobank Japan,^11^ CKD was defined as eGFR of <60 ml/min/1.73 m^2^. In all studies, eGFR was estimated from serum creatinine using the Chronic Kidney Disease Epidemiology Collaboration (CKD-EPI) formula.^15^ The genetic associations with CKD and eGFR were reported in three studies of European ancestry (CKDGen: 41 395 cases, 439 303 controls, 8.7% diabetes patients; UK Biobank: 6985 cases, 454 323 controls, 5.2% with diabetes; and HUNT Study: 3292 cases, 64 476 controls, 4.9% with diabetes). The genetic associations with CKD were reported in three East Asian studies (Biobank Japan: 8586 cases, 133 808 controls, 10.2% with diabetes; China Kadoorie Biobank: 461 cases, 94 887 controls, 6.7% with diabetes; Japan-Kidney-Biobank/ToMMo consortium: 4046 cases, 9423 controls, 7.3% with diabetes) and eGFR genetic associations were reported in Biobank Japan (Supplementary Table S4 and Supplementary Note S2, available as Supplementary data at *IJE* online). All participants included in the CKDGen,^8^ UK Biobank,^9^ HUNT,^10^ Biobank Japan,^11^ China Kadoorie Biobank^12^ and Japan-Kidney-Biobank/ToMMo provided written informed consent and studies were approved by their local research ethics committees and institutional review boards as applicable.

### Statistical analysis

MR is an instrumental variable method that uses genetic variants as instruments to test the causal relationships between an exposure (e.g. BMI) and an outcome (e.g. CKD) and requires three core assumptions to be satisfied (Supplementary Figure S1 and Supplementary Note S3, available as Supplementary data at *IJE* online). For binary exposures [e.g. type 2 diabetes (T2D)], we converted the odds ratios (ORs) [multiplying log(ORs) by log (2) (equal to 0.693) and then exponentiating] in order to represent the OR of outcome per doubling of the odds of susceptibility to the exposure.^16^^,^^17^

The MR estimates for each risk factor were determined using inverse variance weighted (MR-IVW) analysis, which uses the random-effects meta-analysis approach to combine the Wald ratio estimates^18^ of the causal effect obtained from each of the tested SNPs. A set of sensitivity analyses, including MR–Egger,^19^ MR weighted median,^20^ MR mode estimator^21^ and a heterogeneity test,^22^ were conducted to test the underlying MR assumptions. We also examined the possibility of reverse causality using bidirectional MR^23^ and applied multivariable MR analyses of the correlated phenotypes (Supplementary Note S4, available as Supplementary data at *IJE* online). A conservative Bonferroni-corrected threshold (α = 1.11 × 10^–3^, as 45 risk factors were assessed) was used to account for multiple testing. Supplementary Note S5 (available as Supplementary data at *IJE* online) demonstrates the instrument strength estimation^20^ and power calculations.^24^^,^^25^ The MR and sensitivity analyses were conducted using the TwoSampleMR package.^26^

### Follow-up MR analyses

To validate the different causal pattern of blood pressure across ancestries, we conducted a set of follow-up analyses: (i) to estimate the potential influence of instrument size and resulting power of the MR analyses, we conducted novel East Asian GWASs of hypertension (*N* cases = 40 318, *N* controls = 60 323), systolic blood pressure (SBP), diastolic blood pressure (DBP) and pulse pressure (PP) in 100 641 China Kadoorie Biobank participants. By extract genetic instruments from the well-powered GWASs, we further increased the instrument strength (F-statistics) of hypertension from 275.19 to 330.85 (Supplementary Table S3, available as Supplementary data at *IJE* online). Using these data as instruments, we conducted a validation MR between the blood-pressure phenotypes and CKD in the three East Asian studies; (ii) for the European SBP and DBP instruments, we checked whether their genetic associations were replicated in the East Asian GWASs.^27^ We then used the replicated SNPs of SBP and DBP (Supplementary Table S5, available as Supplementary data at *IJE* online) to conduct a second validation MR (noted as European variant + East Asian effect analysis); (iii) we compared the direction of effect and the heterogeneity of the genetic effects of hypertension in Europeans and East Asians using pair-wise Z test and ran sensitivity MR analysis to remove instruments with heterogeneity.

To better understand the causal mechanisms linking T2D with CKD, four additional MR analyses were conducted: (i) we validated the effects of eight glycaemic phenotypes on CKD using Steiger filtering^28^ and radial MR;^29^ (ii) we considered the influence of the genetic liability for type 1 diabetes (T1D)^30^ (Supplementary Table S2, available as Supplementary data at *IJE* online) on CKD; (iii) participants with eGFR measurements were stratified into diabetic (*N* = 11 529) and non-diabetic populations (*N* = 118 460)^31^ and we conducted MR analyses of T2D and five glycaemic phenotypes on eGFR in these two subpopulations; (iv) diabetic retinopathy was included as a positive control outcome to validate the analytical approach. The instruments for T2D and glycaemic phenotypes were used as exposures, whereas the CKD data from CKDGen, UK Biobank and HUNT as well as the diabetic retinopathy data from UK Biobank SAIGE release^32^ were used as outcomes (Supplementary Table S4, available as Supplementary data at *IJE* online).

To validate the MR findings of lipids on CKD, the following analyses were conducted: (i) to validate the high-density lipoprotein cholesterol (HDL-C) MR results in East Asians, we conducted the same European variant + East Asian effect analysis to boost the power of the MR findings (HDL-C data from Spracklen *et al.*^33^) (Supplementary Table S5, available as Supplementary data at *IJE* online); (ii) we tested the independent effect of HDL-C and apolipoprotein A-I on CKD using a multivariable MR model (Supplementary Note S4, available as Supplementary data at *IJE* online); (iii) we estimated the effect of circulating cholesteryl ester transfer protein levels^34^ on CKD (Supplementary Table S2, available as Supplementary data at *IJE* online); (iv) given that lipoprotein(a) levels for a fixed apolipoprotein(a) isoform size may vary, we estimated the effect of apolipoprotein(a) isoform size on CKD [lipoprotein(a) KIV2 repeats and apolipoprotein(a) protein isoform size data from Saleheen *et al.*^35^] (Supplementary Table S2, available as Supplementary data at *IJE* online).

Finally, for BMI and fasting glucose, a fractional polynomial approach^36–38^ was applied to estimate the non-linear shape of the association between these risk factors and CKD using data from UK Biobank and HUNT (Supplementary Note S6, available as Supplementary data at *IJE* online).

### Evaluation of MR evidence

Previous studies have suggested that *P*-value thresholds should not be the only criteria to define ‘significance’.^39^^,^^40^^,^^41^ We therefore evaluated the MR evidence using three criteria: (i) MR evidence strength: whether the MR-IVW estimate of each risk factor passed the Bonferroni-corrected *P*-value threshold (*P* < 1.1 × 10^–3^) in at least one study and passed the replication threshold (*P* < 0.05) in at least one other study; (ii) fit of MR assumptions: whether the MR estimates for each risk factor showed the same direction of effect across MR sensitivity analyses and showed limited influence of horizontal pleiotropy using the MR–Egger intercept term and heterogeneity test; (iii) whether the direction of the MR effect of each risk factor on CKD was consistent across multiple studies. Figure  1 demonstrates how the MR evidence was evaluated in Europeans and East Asians separately: ‘Reliable evidence’ refers to risk factors that fulfilled all the three criteria, whereas ‘Weak evidence’ refers to risk factors that do not fulfil all the criteria (e.g. MR estimates with strong MR evidence but with inconsistent directionality).

## Results

### Causal effects of risk factors on CKD

**Figure 2:**
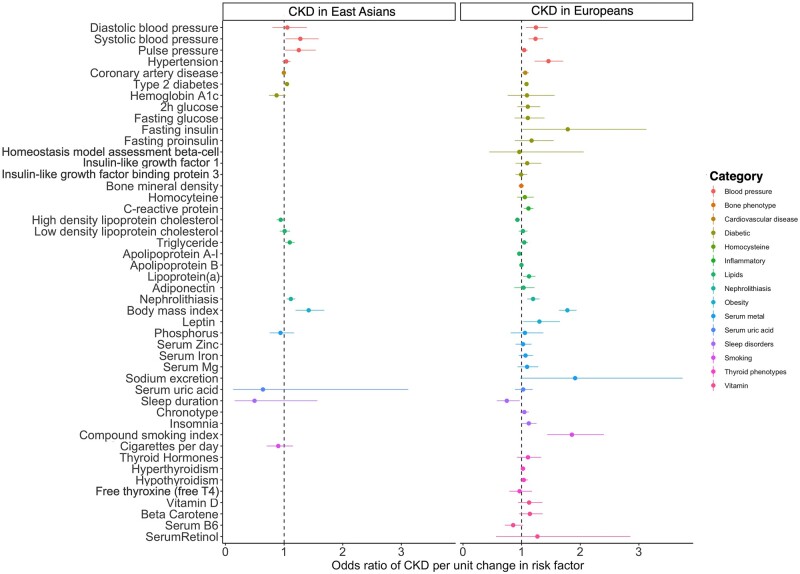
Forest plot for causal effects of the 45 risk factors on chronic kidney disease in Europeans and the 17 risk factors on chronic kidney disease in Eastern Asians. (A) Causal estimates using European data; (B) causal estimates using Eastern Asian data. For binary exposures, the effect reported on the *x*-axis is the odds ratio of chronic kidney disease per doubling in the odds of the exposure. For continuous exposure, the effect on the *x*-axis is the odds ratio of chronic kidney disease per 1 standard deviation change in the exposure. CKD, chronic kidney disease.

Most of the 45 risk factors had strong genetic instruments for both ancestries (F-statistics > 10 for 44 of the 45 risk factors in Europeans and 15 of the 17 risk factors in East Asians; Supplementary Tables S2 and S3, available as Supplementary data at *IJE* online). However, instruments tended to be stronger in Europeans compared with East Asians (Supplementary Table S6, available as Supplementary data at *IJE* online). Effect estimates for the 45 risk factors on CKD in Europeans and 17 risk factors in East Asians are presented in Figure  2. Effect estimates for the other sensitivity analyses can be found in Supplementary Tables S7–S12 (available as Supplementary data at *IJE* online). Detailed evaluations of the causal evidence in Europeans and East Asians are presented in Supplementary Tables S13A and S13B (available as Supplementary data at *IJE* online).

### Risk factors showing reliable MR evidence

In European ancestry, eight risk factors were associated with CKD. The OR [95% confidence intervals (CIs)] for CKD per 1-SD increase in continuous risk factors was 1.78 (1.64 to 1.94) for BMI, 1.24 (1.12 to 1.37) for SBP, 1.13 (1.07 to 1.19) for lipoprotein(a) levels, 0.93 (0.90 to 0.97) for HDL-C and 0.96 (0.94 to 0.98) for apolipoprotein A-I. The OR (95% CI) per doubling in the odds of genetic liability for the binary risk factors was 2.05 (1.59 to 2.64) for hypertension, 1.20 (1.09 to 1.31) for nephrolithiasis and 1.08 (1.05 to 1.12) for T2D. The effects of these eight risk factors on CKD were consistent across UK Biobank, CKDGen and HUNT (Supplementary Figure S2A and Supplementary Tables S7A, S8A and S9A, available as Supplementary data at *IJE* online).

In East Asian participants, genetically predicted higher BMI (OR = 1.42, 95% CI = 1.20 to 1.69, *P* = 6.49 × 10^–5^), increased nephrolithiasis risk (OR = 1.12, 95% CI = 1.04 to 1.19, *P* = 1.11 × 10^–3^) and increased T2D risk (OR = 1.07, 95% CI = 1.03 to 1.10, P = 1.66 × 10^–4^) were all associated with increased risk of CKD (Supplementary Figure S2B, available as Supplementary data at *IJE* online). The effect of T2D on CKD was consistent across the three East Asian studies. However, the effect of BMI and nephrolithiasis on CKD was not observed in the China Kadoorie Biobank—this is likely due to the limited number of CKD cases in this resource (Supplementary Tables S10A–S12, available as Supplementary data at *IJE* online).

We also conducted several sensitivity analyses. The bidirectional MR analysis found a consistent effect of increased CKD risk on increasing hypertension risk in European ancestry (Supplementary Table S14, available as Supplementary data at *IJE* online). The multivariable MR results for T2D, BMI and hypertension on CKD are included in Supplementary Table S15A–C, available as Supplementary data at *IJE* online. The MR analyses using eGFR as an outcome showed similar results to those for CKD (Supplementary Tables S7B–S10B, available as Supplementary data at *IJE* online).

### Risk factors showing weak MR evidence

There was weak evidence to support a causal effect on CKD of the remaining 37 risk factors considered in the European ancestry analyses (Supplementary Tables S7–S9, available as Supplementary data at *IJE* online) and 14 risk factors considered in the East Asian ancestry analyses (Supplementary Tables S10–S12, available as Supplementary data at *IJE* online). Some established risk factors, such as smoking and serum uric acid, were among those with weak evidence. In addition, shorter sleep duration showed evidence to support an association with CKD in Japan-Kidney-Biobank/ToMMo (Supplementary Table S11, available as Supplementary data at *IJE* online) and in UK Biobank (Supplementary Table S7A, available as Supplementary data at *IJE* online), which was not replicated in other studies.

### Follow-up MR analyses of key findings

#### Effect of blood-pressure phenotypes on CKD across populations

**Figure 3:**
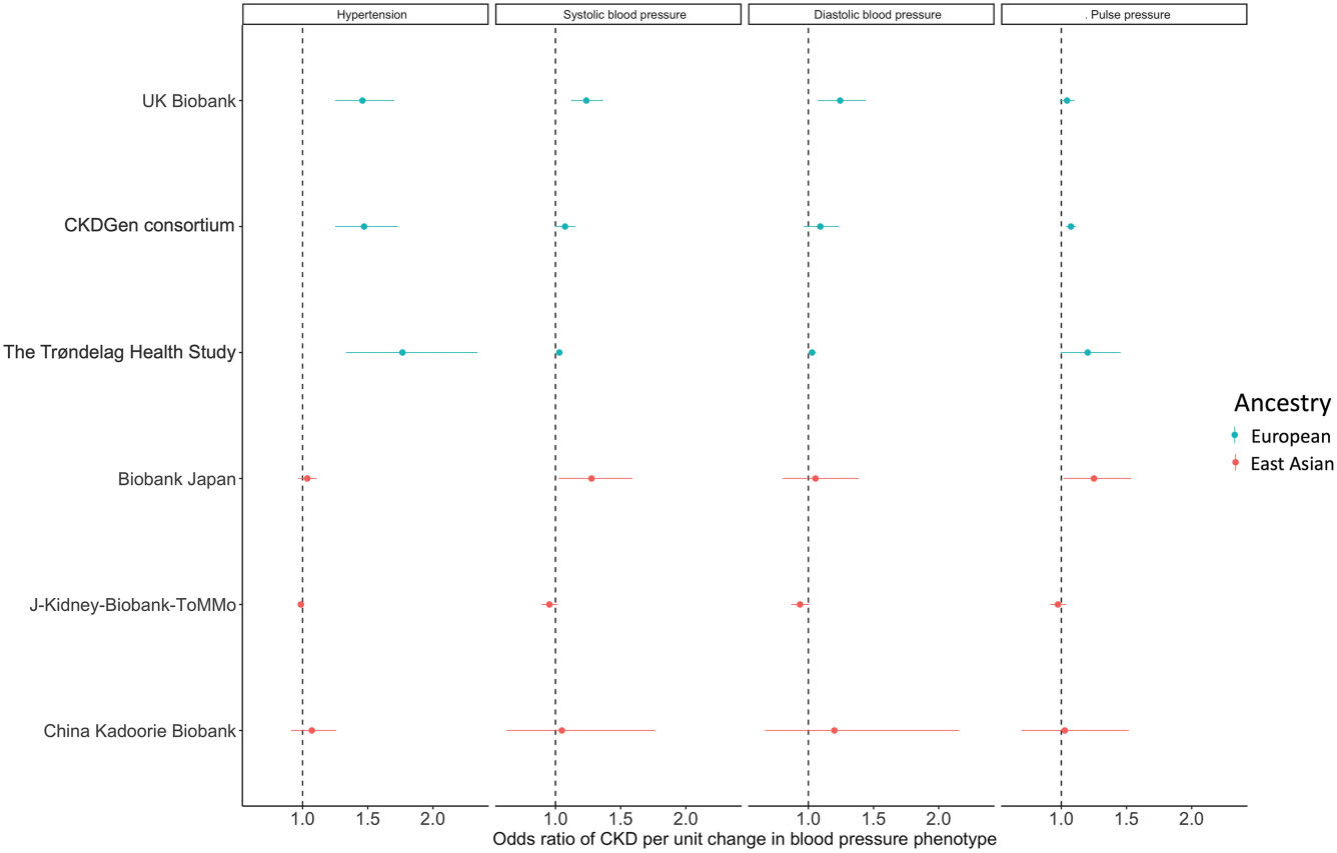
Forest plot for causal effects of four blood-pressure phenotypes on chronic kidney disease risk. The subplots represent Mendelian-randomization results of different blood-pressure phenotypes. CKD, chronic kidney disease.

Figure  3 demonstrates that blood-pressure phenotypes, including genetic liability of hypertension and genetically predicted SBP and PP, showed strong causal effects on CKD in the European studies but appeared to show a null causal effect in the corresponding East Asian studies (ORs for liability of hypertension on CKD ranging from 1.46 to 1.77 in Europeans but only from 0.99 to 1.07 in East Asians). To validate these MR results, we first checked the strength of genetic instruments for the four blood-pressure phenotypes and observed that the instrument strengths were substantially above the F-statistics threshold of 10 for all four phenotypes in Europeans and East Asians (Supplementary Table S6, available as Supplementary data at *IJE* online). To further boost power, we used genetic instruments for hypertension, SBP, DBP and PP from 100 641 China Kadoorie Biobank participants [which obtained better instrument strength than the European hypertension data (F-statistics = 61.11 in Europeans vs 330.85 in East Asians); Supplementary Tables S2 and S3, available as Supplementary data at *IJE* online] and still observed null results in East Asians (Supplementary Table S16A, available as Supplementary data at *IJE* online). Third, we conducted an MR analysis using the European SBP and DBP instruments extracted from the East Asian studies (Supplementary Table S5, available as Supplementary data at *IJE* online), which showed similar null results (Supplementary Table S16B, available as Supplementary data at *IJE* online). Finally, we estimated the heterogeneity of genetic effects of hypertension across Europeans and East Asians and observed that 20.9% of the instruments showed distinguished effects across the two ancestries (Supplementary Table S17, available as Supplementary data at *IJE* online). Sensitivity MR analyses excluding the heterogenous instruments, controlling for different genetic architectures of BP across ancestries, showed similar MR results (Supplementary Table S18, available as Supplementary data at *IJE* online). These analyses provide additional evidence that blood pressure has a population-specific role in CKD aetiology.

#### Effects of glycaemic phenotypes and CKD

Although the evidence for an effect of T2D on CKD was reliable, we detected little evidence to support the effects of eight glycaemic phenotypes [fasting insulin (FI), fasting glucose (FG), 2-hour glucose (2hGlu), fasting proinsulin (FP), haemoglobin A1c (HbA1c), HOMA-B, insulin-like growth factor binding protein 3 and insulin-like growth factor I] on CKD (Supplementary Figure S3, available as Supplementary data at *IJE* online) and eGFR (Supplementary Figure S4, available as Supplementary data at *IJE* online). Follow-up analyses showed that: (i) similar results were observed after controlling for possible reverse causation of instruments and potential outliers (Supplementary Table S19A, available as Supplementary data at *IJE* online); (ii) little evidence was observed that genetic liability to T1D was associated with CKD risk in any of the three outcome studies from European ancestry (Supplementary Table S19B, available as Supplementary data at *IJE* online), which further supported the weak effect of glucose on CKD; (iii) for the MR analysis using stratified eGFR in Europeans, little effect of glycaemic phenotypes on eGFR was observed in both diabetic and non-diabetic samples (Supplementary Table S19C, available as Supplementary data at *IJE* online), which suggested that the weak effect of glucose on CKD could be independent of diabetes; (iv) fasting glucose and genetic liability to T2D were associated with diabetic retinopathy (Supplementary Table S19D, available as Supplementary data at *IJE* online), suggesting that the genetic predictors of glycaemic phenotypes used for the main MR analyses were reliable.

#### Effects of blood lipids and CKD

For the MR findings of lipids, our follow-up analyses showed a few novel observations. First, we observed different MR evidence for genetically predicted HDL-C on CKD across Europeans and East Asians. In Europeans, good MR evidence was observed to support the effects of HDL-C and apolipoprotein A-I on CKD (Supplementary Tables S7–S9, available as Supplementary data at *IJE* online), whereas there was weaker MR evidence for the effect of HDL-C on CKD in East Asians (Supplementary Tables S10–S12, available as Supplementary data at *IJE* online). To test the potential influence of the power of the HDL-C effect on CKD in East Asians (OR = 0.94, 95% CI = 0.87 to 1.02), we conducted MR using better-powered European HDL-C instruments extracted from the East Asian studies (Supplementary Table S5, available as Supplementary data at *IJE* online). Using this approach, we found reliable MR evidence (OR = 0.89, 95% CI = 0.83 to 0.96; Supplementary Table S16C, available as Supplementary data at *IJE* online). This suggests that HDL-C may have an effect on CKD in both populations. Second, using European data, a multivariable MR considering both HDL-C and apolipoprotein A-I in the same model was conducted. This showed that the effect of HDL-C on CKD was independent of apolipoprotein A-I (Supplementary Table S15D, available as Supplementary data at *IJE* online). Third, following the HDL-C finding, we found an effect of the circulating cholesteryl ester transfer protein level on CKD in CKDGen (OR = 1.06, 95% CI = 1.01 to 1.11, *P* = 0.01; Supplementary Table S20, available as Supplementary data at *IJE* online). Finally, we investigated the potential influence of the apolipoprotein(a) size on CKD but found little evidence for a causal effect. This suggests that the effect of the lipoprotein(a) level on CKD may be independent of the apolipoprotein(a) size (Supplementary Table S21, available as Supplementary data at *IJE* online).

#### Non-linear effects of BMI and fasting glucose on CKD

**Figure 4:**
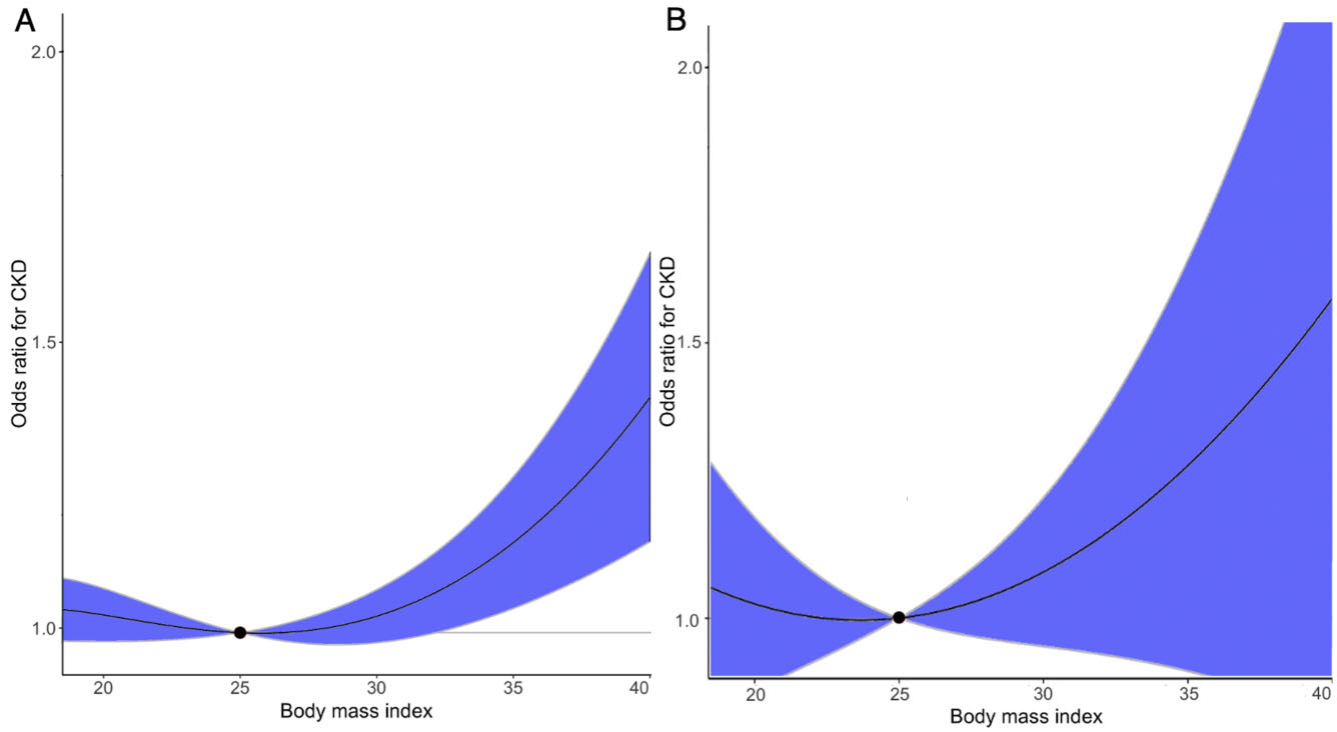
Non-linear Mendelian randomization of body mass index on chronic kidney disease risk. The dose–response curve between body mass index and chronic kidney disease risk for (A) UK Biobank and (B) the HUNT Study. The gradient at each point of the curve is the localized average causal effect. Shaded areas represent 95% confidence intervals. CKD, chronic kidney disease.

We observed a threshold relationship between genetically predicted BMI and CKD (Supplementary Table S22, available as Supplementary data at *IJE* online). The curved shape of this relationship suggests a higher risk of CKD in overweight or obese participants, with the optimal BMI threshold at ∼25 kg/m^2^ in both UK Biobank and HUNT (Figure  4). Stratified analyses split by sex (Supplementary Figure S5, available as Supplementary data at *IJE* online) and age (Supplementary Figure S6, available as Supplementary data at *IJE* online) suggested similar effects for genetically predicted BMI on CKD. Genetically predicted fasting glucose showed weak evidence for a non-linear relationship with CKD (Supplementary Figure S7 and Supplementary Table S22, available as Supplementary data at *IJE* online). This finding was consistent both among males and females (Supplementary Figure S8, available as Supplementary data at *IJE* online) and different age groups (Supplementary Figure S9, available as Supplementary data at *IJE* online).

As a summary, we systematically compared the MR findings with existing clinical evidence in KDIGO guidelines and listed the potential clinical implications in Table  1.

**Table 1:** Systematic evaluation of Mendelian-randomization evidence with existing clinical evidence

Risk factors	Clinical evidence	MR evidence	Clinical implications
	in KDIGO guideline	from this study	
Blood pressure	Established	Strong evidence in Europeans	Suggest different prevention strategy for CKD across ancestries
		Weak evidence in East Asians	
BMI	Emerging	Strong in both ancestries	Suggest optimal control level of BMI as 25
Nephrolithiasis	Emerging	Strong in both ancestries	Suggest assessment of kidney stones in high-risk groups
Diabetic phenotypes	Established	Strong for diabetes but weak evidence for other glycaemic traits	Imply glucose-independent effect of diabetes on CKD
Lipid phenotypes	Established	Strong evidence for HDL-C, cholesteryl ester transfer protein (CETP) and Lp(a)	Suggest cholesteryl ester transfer protein (CETP) and Lp(a) inhibition as intervention targets for CKD prevention
Low sleep duration	Emerging	Strong evidence in one study but lack of replication in other studies	Suggest future studies to confirm the effect of sleeping on CKD
Smoking, uric acid, CRP, bone, metal, vitamin, thyroid phenotypes	Emerging	Little evidence	–

CKD, chronic kidney disease; BMI, body mass index; HDL, high-density lipoprotein; CETP, cholesteryl ester transfer protein; Lp(a), lipoprotein (a); CRP, C-reactive protein.

## Discussion

In this trans-ethnic MR study, we comprehensively assessed the causality of 45 risk factors on CKD and eGFR in >1 million Europeans and 17 risk factors on CKD and eGFR in >250 000 East Asians. Using MR approaches, including five two-sample MR methods and multivariable MR, we found reliable evidence for the causal effects of eight cardiometabolic-related risk factors [BMI, SBP, hypertension, T2D, nephrolithiasis, HDL-C, apolipoprotein A-I and lipoprotein(a)] on CKD. The remaining 37 risk factors, including smoking and serum uric acid, had weak evidence to support causal effects on CKD using the currently available data. These findings are consistent with previous MR studies that analysed similar risk factors separately.^40–44^ In addition, the null finding of the serum uric acid agreed with the recent clinical trial investigating the effects of serum urate lowering (using Allopurinol) on CKD progression.^45^^,^^46^ Notably, our extensive MR and follow-up analyses suggested the possibility of glucose-independent pathways linking T2D with CKD. Using non-linear MR, we observed a threshold relationship between genetically predicted BMI and CKD risk, with increased CKD risk at a BMI of >25 kg/m^2^.

The causal patterns of 17 risk factors were compared across the two ancestries and we observed consistent effects of T2D, BMI and nephrolithiasis on CKD in Europeans and East Asians. In contrast, distinguishable causal patterns between ancestries were observed when examining the effect of hypertension on CKD, with a strong causal estimate in Europeans that was not replicated in the analysis of East Asians. These findings indicate that careful consideration is needed before implementing interventions for CKD risk factors in participants of one ancestry based on the evidence from another ancestry.

Among the prioritized risk factors, hypertension is one of the most common risk factors for kidney-function decline in patients with or without CKD.^47–49^ A recent bidirectional MR study in Europeans supported the causal effects of higher kidney function on lower blood pressure using eGFR instruments controlled by blood urea nitrogen. However, the same study suggested inconclusive evidence of an effect of blood pressure on eGFR.^50^ In our MR analysis, we found evidence of positive bidirectional causal effects between hypertension and CKD in Europeans. There are several potential explanations for the inconsistent MR findings across these studies. Yu *et al.* used genetic associations for blood pressure that were adjusted for BMI with genetic associations for eGFR and CKD that were not adjusted for BMI in their MR analysis. Given the causal role of BMI on both CKD and hypertension, only controlling for BMI in the exposure data may create unintended bias in the MR estimates, as described previously.^51^ An alternative explanation is the difference in CKD case ascertainment. Specifically, we used CKD cases that were clinically diagnosed, which may bring additional statistical power and provide more reliable evidence for the effect of blood pressure on CKD.

Given the difference in MR evidence across the ancestries that we observed, combined with previous evidence from the literature, it is possible that hypertension could have differential effects on CKD by ancestry. Ethnic disparities in relation to hypertension and CKD have previously been reported.^52^^,^^53^ For example, Chinese people with hypertension have a lower risk of CKD compared with European people with hypertension.^52^ Additionally, in 2019, hypertensive nephropathy accounted for 27% of the overall CKD cases in the USA but 20.8% of the overall CKD cases among Chinese.^54^^,^^55^ Further well-powered studies are needed to validate the causal effect of blood pressure on CKD across ancestries.

In addition, our MR analyses suggested substantial causal effects for BMI and nephrolithiasis on CKD. Previous observational studies have suggested that BMI is positively associated with CKD onset^56^ and end-stage renal disease^57^^,^^58^ and negatively associated with kidney function.^59^ The effect of weight loss on reducing the risk of diabetic nephropathy in patients with T2D^60^ and slowing kidney-function decline have also been reported.^61^ Using linear and non-linear MR approaches, we observed a threshold causal relationship between BMI and CKD. Moreover, nephrolithiasis is a common and serious health concern globally.^62–64^ There is increasing evidence to suggest that having kidney stones is a risk factor for CKD.^62^^,^^65^ For instance, people with kidney stones tend to have lower eGFR.^63^^,^^66^ A previous cohort study suggested that even a single kidney-stone episode was associated with an increased likelihood of adverse renal outcomes.^67^ A recent genetic study also suggested an inverse association between eGFR and kidney-stone formation.^68^ However, the causal relationship between nephrolithiasis and CKD had not been investigated previously. Our MR analysis supported the causal effect of increased nephrolithiasis risk on increasing CKD risk. This is of particular importance as obstructive nephropathy is among the leading causes of CKD in the general population. Specifically, it is the third leading cause of CKD among the Chinese population and has been estimated to be present in 15.6% of CKD cases.^55^

Notably, diabetic kidney disease is considered the most common type of CKD worldwide.^69^ A previous MR study of T2D on CKD in Chinese participants suggested a strong causal link between the two phenotypes,^70^ which aligned with our MR findings in both East Asians and Europeans. However, despite the reliable evidence for a causal effect of T2D on CKD, our linear and non-linear MR found limited evidence to support the causal effects of glucose- and insulin-related phenotypes on CKD. This is consistent with the findings from a previous MR study conducted in Europeans.^71^ It has also been observed that with increasing use of glucose-lowering medications, the prevalence of CKD in diabetics has not reduced as much as expected.^72^ This is supported by a meta-analysis of RCTs that found intensive glucose control to have an inconclusive effect on reducing the risk of end-stage renal disease.^73^ These findings, together with our MR results, suggest that glucose-independent pathways could play a role in the relationship between diabetes and CKD. Furthermore, it has consistently been suggested that the beneficial effects of SGLT2 inhibitors (antidiabetic medication) on renal outcomes may be mediated by glucose-independent pathways.^74^^,^^75^ One potential limitation of our analysis in relation to interrogating this finding is that the glucose GWAS that we used was conducted in a general population whose fasting glucose levels are <7 mmol/L. Existing MR studies using these data have made the assumption that the glucose change in the general population is similar to that in diabetic patients (i.e. individuals whose fasting glucose levels are typically >7 mmol/L), which may not necessarily be true. Although our stratified MR analysis showed little difference between diabetic and non-diabetic patients, we believe that better genetic instruments derived from a diabetic patient population and well-designed clinical trials are needed to evaluate the effect of glucose-dependent and -independent mechanisms on CKD prevention.

Hyperlipidaemia and dyslipidaemia have been widely documented to be associated with kidney disease.^76^^,^^77^ But the causal effects of lipid components on CKD are still unclear. A few recent MR studies have suggested a protective effect of higher HDL-C on CKD in Europeans,^78^ an adverse effect of higher triglycerides on CKD in Chinese^79^ and a nominal effect of lipoprotein(a) lowering on reducing CKD risk.^80^ In this study, we validated the HDL-C findings, confirmed the triglycerides effect in Biobank Japan and strengthened the evidence of the lipoprotein(a) finding in completely independent samples. Besides confirming these existing findings, our study also established novel causal evidence for the apolipoprotein A-I and non-apolipoprotein A-I properties of HDL-C on CKD risk in Europeans. Furthermore, our study extended the findings from recent studies of HDL-C^81^ and cholesteryl ester transfer protein inhibitors,^82^ which support the causal effect of circulating cholesteryl ester transfer protein levels on CKD in Europeans. The observed causal effect of HDL-C and the effect of cholesteryl ester transfer protein levels on CKD raises the possibility that increasing the HDL-C concentration may offer a potential intervention strategy for CKD prevention. Moreover, our study demonstrated that the causal effect of lipoprotein(a) levels on CKD was independent of the apolipoprotein(a) size. This finding, together with previous observational evidence,^83–85^ implies the possibility of lipoprotein(a)-reduction therapies, such as Pelacarsen [also known as IONIS-APO(a)-L_Rx_], on reducing CKD risk.^86^ Overall, our findings have highlighted the potential for several lipid-management strategies in reducing CKD risk.

### Strengths and limitations

Our study has some strengths compared with previous studies in this setting. We used clinically diagnosed CKD (instead of only using eGFR < 60 ml/min/1.73 m^2^ to define CKD) in two European (UK Biobank and HUNT) and two East Asian (China Kadoorie Biobank and Japan-Kidney-Biobank/ToMMo) studies. These four studies included participants with abnormal urine protein levels but with normal eGFR as CKD cases. This increased the robustness of the CKD definition. By comprehensively validating the MR findings in the six CKD studies, we also greatly enhanced the reliability of the causal atlas that we derived of risk factors for CKD.

Our study also has some potential limitations. First, we used the ICD 10 code to define CKD cases in three of the six studies. Such selection criteria excluded undiagnosed cases and diagnoses made in an outpatient setting. Considering the low disease awareness of CKD,^87^^,^^88^ such misclassification of the outcome may reduce the power of our study. However, as a trade-off, such an approach also excluded non-CKD samples from the case group (e.g. participants with a single eGFR measurement of <60 due to measurement error), which brought additional power to the statistical analysis. Second, we set up a stringent Bonferroni-corrected threshold together with other criteria (e.g. little evidence of pleiotropy) to select the top MR findings. Such a strategy could create some false-negative findings but minimize the possibility of identifying false-positive findings. With the aim of supporting the future clinical practice of CKD management, we decided to apply such a stringent strategy to provide the most reliable causal evidence using genetics. Second, in the MR analysis, genetic predictors for binary exposures (e.g. coronary artery disease) are not mimicking the exposure itself, but the predisposition to the exposure instead.^89^ Consequently, our results must be interpreted as the effect of removing the predisposition to the binary exposure (rather than treatment of the exposure) to reduce CKD risk. In addition, due to the relative lack of GWAS samples in East Asians, we could only examine causal effects for 17 of the 45 risk factors for this ancestry. For the same reason, the number of instruments for each risk factor in the analyses differed between the two ancestries. For risk factors with different MR evidence across ancestries, we conducted a comprehensive set of sensitivity analyses to minimize the influence of differences in power and instrument strength across ancestries. Other limitations of the study are listed in Supplementary Note S7 (available as Supplementary data at *IJE* online).

## Conclusions

By evaluating the causal evidence for 45 risk factors on CKD in >1 million individuals of European ancestry and 17 risk factors in >250 000 individuals of East Asian ancestry, we have shown that eight risk factors are reliably causal for CKD in Europeans and three of these are also causal in East Asians. These risk factors are predominantly related to cardiometabolic health, which supports the shared causal link between cardiometabolic health and kidney function. The different causal pattern between hypertension and CKD in Europeans compared with that in East Asians suggests that blood pressure might have an ancestry-specific role in CKD aetiology. Ultimately, our findings may have important clinical implications in terms of informing primary prevention in ‘at-risk’ individuals with normal renal function, which may in turn help to reduce the burden of CKD globally.

## Supplementary data

Supplementary data are available at *IJE* online.

## Ethics approval

The HUNT study was approved by the Central Norway Regional Committee for Medical and Health Research Ethics (REC Central no. 2015/1188) and written informed consent was given by all participants. The UK Biobank study has ethical approval from the North West Multicentre Research Ethics Committee (MREC). The China Kadoorie Biobank study obtained ethics approval from the Oxford University Tropical Research Ethics Committee (approval number: 025–04, 3.2.2005), the Chinese Centre for Disease Control and Prevention (CDC) Ethical Review Committee (approval number: 005/2004, 9.7.2004) and the local CDC of each study area.

## Funding

This research has been conducted using the UK Biobank resource under Application Numbers ‘40135’ and ‘15825’. J.Z. is funded by a Vice-Chancellor Fellowship from the University of Bristol. This research was also funded by the UK Medical Research Council Integrative Epidemiology Unit [MC_UU_00011/1, MC_UU_00011/4 and MC_UU_00011/7]. J.Z. is supported by the Academy of Medical Sciences (AMS) Springboard Award, the Wellcome Trust, the Government Department of Business, Energy and Industrial Strategy (BEIS), the British Heart Foundation and Diabetes UK [SBF006\1117]. This study was funded/supported by the NIHR Biomedical Research Centre at University Hospitals Bristol NHS Foundation Trust and the University of Bristol (G.D.S., T.R.G. and R.E.W.). This study received funding from the UK Medical Research Council [MR/R013942/1]. J.Z., Y.M.Z. and T.R.G are funded by a BBSRC Innovation fellowship. J.Z. is supported by the Shanghai Thousand Talents Program. Y.M.Z. is supported by the National Natural Science Foundation of China [81800636]. H.Z. is supported by the Training Program of the Major Research Plan of the National Natural Science Foundation of China [91642120], a grant from the Science and Technology Project of Beijing, China [D18110700010000] and the University of Michigan Health System–Peking University Health Science Center Joint Institute for Translational and Clinical Research [BMU2017JI007]. N.F. is supported by the National Institutes of Health awards R01-MD012765, R01-DK117445 and R21-HL140385. R.C. is funded by a Wellcome Trust GW4 Clinical Academic Training Fellowship [WT 212557/Z/18/Z]. The Trøndelag Health Study (the HUNT Study) is a collaboration between HUNT Research Centre (Faculty of Medicine and Health Sciences, NTNU, Norwegian University of Science and Technology), Trøndelag County Council, Central Norway Regional Health Authority and the Norwegian Institute of Public Health. M.C.B. is supported by the UK Medical Research Council (MRC) Skills Development Fellowship [MR/P014054/1]. S.F. is supported by a Wellcome Trust PhD studentship [WT108902/Z/15/Z]. Q.Y. is funded by a China Scholarship Council PhD scholarship [CSC201808060273]. Y.C. was supported by the National Key R&D Program of China [2016YFC0900500, 2016YFC0900501 and 2016YFC0900504]. The China Kadoorie Biobank baseline survey and the first resurvey were supported by a grant from the Kadoorie Charitable Foundation in Hong Kong. The long-term follow-up is supported by grants from the UK Wellcome Trust [202922/Z/16/Z, 088158/Z/09/Z and 104085/Z/14/Z]. Japan-Kidney-Biobank was supported by AMED under Grant Number 20km0405210. P.C.H. is supported by Cancer Research UK [grant number: C18281/A19169]. A.K. was supported by DFG KO 3598/5–1. N.F. is supported by NIH awards R01-DK117445, R01-MD012765 and R21-HL140385. The views expressed in this publication are those of the author(s) and not necessarily those of the NHS, the National Institute for Health Research or the Department of Health.

## Data availability

The genetic-association data of the selected risk factors are available in Supplementary Tables (available as Supplementary data at *IJE* online). The GWAS summary statistics for CKD and eGFR that were generated using UK Biobank and CKDGen data are available from the MRC-IEU OpenGWAS database (https://gwas.mrcieu.ac.uk/) and CKDGen website (http://ckdgen.imbi.uni-freiburg.de/), respectively. The GWAS results from HUNT, Biobank Japan, China Kadoorie Biobank and Japan-Kidney-Biobank/ToMMo can be accessed by request to the data holders. The raw China Kadoorie Biobank data underlying this article can be accessed via a formal data request to ckbaccess@ndph.ox.ac.uk, following the institution’s data-access policies. Preliminary event adjudication data are not publicly available. The data underlying this article will be shared on reasonable request to the corresponding author.

## Author contributions

J.Z. is the guarantor; J.Z., Y.M.Z. and H.R. performed the linear MR analysis; J.Z. and H.R. performed the non-linear MR with support from S.F., Q.Y. and S.B.; J.Z., H.R. and L.F.T. performed the GWAS in UK Biobank and the HUNT Study; Y.S., M.Y. and N.K. conducted the GWAS in each cohort and performed the GWAS meta-analysis in the Japan-Kidney-Biobank/ToMMo study; M.A., M.K., K.M., Y.K. and Y.O. performed the GWAS in Biobank Japan; C.Q.Y. and J.C.L. conducted the GWAS in the China Kadoorie Biobank; J.Z., Y.M.Z. and B.E. performed the systematic review of CKD risk factors; R.E.W. performed the sensitivity analyses of smoking and CKD; P.C.H., A.H., J.R., B.M.B., L.F.T., K.H., S.H., A.K., C.P., M.W. and B.O.A. provided key data and supported the MR analyses; M.C.B., Y.C., R.C., S.H., N.F., A.P.M., G.D.S., S.B., C.Q.Y. and B.O.A. reviewed the paper and provided key comments; J.Z., Y.M.Z., H.R., V.W., Y.S., Y.L., G.D.S., S.B., B.O.A., H.Z. and T.R.G. wrote the manuscript; J.Z., Y.M.Z., H.Z. and T.R.G. conceived of and designed the study and oversaw all analyses.

## Conflict of interest

T.R.G. and J.Z. receive funding from GlaxoSmithKline and Biogen for unrelated research. None declared by the other authors.

## Supplementary Material

dyab203_Supplementary_Data
