# Supplementary material for: Trans-ethnic Mendelian-randomization study reveals causal relationships between cardiometabolic factors and chronic kidney disease
Source: Int J Epidemiol. 2021 Oct 20;50(6):1995–2010. doi: 10.1093/ije/dyab203 (PMC8743120; doi:10.1093/ije/dyab203)
Supplement: dyab203_Supplementary_Data [file dyab203_supplementary_data.zip › ije-2021-06-0996-File007.docx]

# Supplementary Notes

## Note S1. Identification of previously reported risk factors and instrument selection

We conducted the literature review using text mining approaches and further mapped the reported risk factors to the largest genetic association studies in order to select genetic instruments for the risk factors.

The literature information was obtained from SemMedDB, implemented in MELODI Presto (1)(2), which summarizes the literature information as “subject-predicate-object” triples. The subject is the risk factor of interest. The object is the phenotype of interest – for instance, “chronic kidney disease”. The predicate is a term linking the subject and object together, where the term “CAUSES” implies causality, the term “ASSOCIATED_WITH” implies association, and the term “COEXISTS_WITH” implies co-existence. The enrichment odds ratio and P value for each triple was estimated based on the number of appearances in the literature (e.g. the number of PubMed IDs supporting the existence of the triple). We implemented the following procedure to select the most reliable risk factors for CKD:

1. We selected triples with an enrichment P value < 0.005 (this is the default threshold and suggests good coverage in the literature).
2. We removed triples where the subject was a gene or protein, i.e. triples where the subject may not be considered a risk factor.
3. We retained only unique risk factors (subjects) to avoid duplicates.
4. We restricted to triples where the object contained either “kidney” or “renal”.

MELODI Presto identified a total of 86 risk factors using this approach (**Table S23**). Further filtering for very similar risk factors (e.g, Cardiovascular Diseases and Cardiovascular morbidity) reduced the list to 42 unique risk factors.

Next, we selected genetic instruments to represent each of the 42 unique risk factors from existing GWASs (**Table S24**). Six of the 42 risk factors did not have sufficiently strong genetic instruments for the Mendelian randomization (MR) analyses (no genetic variants associated with the risk factor at P < 5×10^-8^) and were removed from the risk factors list. For the remaining 36 risk factors, six risk factors (Table below) were studied using multiple (n=16) matching phenotype definitions from the GWAS literature.

| MELODI Presto identified risk factor | Matching GWAS phenotypes | Number of risk factors included in the MR |
| --- | --- | --- |
| Hypertensive disease | Diastolic blood pressure  Systolic blood pressure  Pulse pressure  Hypertension | 4 |
| Sleep disorders | Chronotype  Sleep duration  Insomnia | 3 |
| Insulin | Fasting insulin  Fasting proinsulin  HOMA-B | 3 |
| Glucose intolerance | Fasting glucose  2-hour glucose | 2 |
| Hypervitaminosis A | Serum retinol  Beta carotene | 2 |
| Cigarette smoke | Compound smoking index (in Europeans)  Cigarettes per day (in East Asians) | 1 (in each ancestry) |

The other 30 risk factors had one matching phenotype GWAS each. Thus, we considered a total of 45 risk factors in the MR analyses of Europeans. In East Asians, we were only able to find GWAS for 17 of these 45 risk factors.

In summary, the European datasets included all 45 risk factors: four blood pressure phenotypes (systolic blood pressure [SBP], diastolic blood pressure [DBP], hypertension, and pulse pressure [PP]), nine diabetes related phenotypes (Type 2 diabetes [T2D], hemoglobin A1c [HbA1c], 2-hour glucose, fasting glucose, fasting insulin, fasting proinsulin, HOMA-B, Insulin-Like Growth Factor Binding Protein 3, Insulin-Like Growth Factor I), compound smoking index, three sleeping phenotypes (chronotype, sleep duration and insomnia), body mass index (BMI), leptin, nephrolithiasis, serum uric acid, seven blood lipid phenotypes (high density lipoprotein cholesterol [HDL-c], low density lipoprotein cholesterol [LDL-c], triglyceride, adiponectin, apolipoprotein A-I, apolipoprotein B, lipoprotein[a]), coronary artery disease, bone mineral density, homocysteine, C-reactive protein, serum Mg, sodium excretion, phosphorus, serum iron, serum zinc, Vitamin A (serum retinol and beta carotene), Vitamin D, Vitamin B6, hypothyroidism, thyroid hormones, FT4, hyperthyroidism (3)(4)(5)(6)(7) (8)(9)(10)(11)(12)(13)(14)(15)(16)(17)(18)(19)(20)(21)(22)(23)(24)(25)(26)(27)(28)(29)(30)(31)(32)(33)(34)(35)(36). Of the 45 risk factors, 17 can be derived from East Asian GWASs, including SBP, DBP, PP, hypertension, T2D, HbA1c, cigarettes per day, chronotype, BMI, nephrolithiasis, serum uric acid, HDL-c, LDL-c, triglyceride, coronary artery disease, C-reactive protein, and phosphorus (37)(38)(39)(40)(41)(42)(43) (44)(45)(46)(47)(48).

## Note S2. Detailed information of the CKD outcome studies

For European samples, we first conducted two novel GWASs in the UK Biobank and HUNT. In UK Biobank, we performed GWASs of CKD (binary variable, N-cases= 6,985, N-controls= 454,323; 5.2% diabetes patients, 13.8% CKD cases with diabetes) and eGFR (continuous variable, N= 464,207). The CKD cases were defined as ICD 10 code N18, and controls were other participants who had no record of ICD 10 codes N00-N29 (other kidney conditions). eGFR was estimated using the Chronic Kidney Disease Epidemiology Collaboration (CKD-EPI) equation (49) after excluding subjects with creatinine values of <0.4 mg/dl. The GWAS was performed using BOLT-LMM (50) with linear mixed models and an additive genetic model adjusted for age, sex, genotyping array, and genetic correlation matrix (https://doi.org/10.5523/bris.pnoat8cxo0u52p6ynfaekeigi).

In HUNT, we identified 67,768 subjects of European ancestry for our study. Genetic association analyses were conducted for CKD (binary variable N-cases= 3,292, N-controls= 64,476, 4.9% diabetes patients, 10.7% CKD cases with diabetes), BMI (kg/m^2^), HDL (mmol/L), SBP and DBP (mmHg). Age and sex were included in the linear and logistic regression models as covariates. The SNP associations were standardized to Standard Deviation (SD) units after the genetic association analysis to allow direct comparison of MR results across different phenotypes. The HUNT Study recruited from the adult population of the demographically stable Nord-Trøndelag region, Norway, as detailed elsewhere (51). So far, four health surveys have been conducted, HUNT1 (1984–1986), HUNT2 (1995–1997), HUNT3 (2006–2008) and HUNT4 (2017-2019) (<https://www.ntnu.edu/hunt>). Using the ICD 10 code N18 from the hospital registry to define events, we identified 3,292 CKD cases and 64,476 controls (others who have no ICD 10 codes N00-N29). DNA from these subjects was previously genotyped (51) using one of the three different Illumina HumanCoreExome arrays: HumanCoreExome12 v1.0, HumanCoreExome12 v1.1 and UM HUNT Biobank v1.0. Quality control of the genotypes was performed separately for each array and the call rate of genotyped samples was >99%. Imputation was performed on samples of recent European ancestry using Minimac3 (v2.0.1, <http://genome.sph.umich.edu/wiki/Minimac3>) (52) from a merged reference panel constructed from i) the Haplotype Reference Consortium panel (release version 1.1) (53) and ii) a local reference panel based on 2,202 whole-genome sequenced HUNT participants (51).

As the third European dataset, we utilized existing GWAS summary statistics for CKD and eGFR from the CKDGen consortium (54). These two GWASs were meta-analyses of 23 CKD studies (N-cases= 41,395; N-controls= 439,303; 8.7% diabetes patients, 11.1% CKD cases with diabetes) and 42 eGFR studies (N= 567,460). The serum creatinine was measured at baseline for all studies. eGFR was calculated using the CKD-EPI equation. CKD was defined as eGFR < 60 ml/min/1.73m^2^. Genetic associations were adjusted for sex, age and study specific features, including study sites and genetic principal components, and family-based studies were included with consideration of relatedness (55).

For East Asian samples, we utilized GWAS summary statistics of CKD (N-case= 8,586, N-control=133,808) from Biobank Japan, J-Kidney-Biobank/TOMMO (N-case=4,046, N-control=9,423) and China Kadoorie Biobank (N-case=848, N-control=94,887), as well as eGFR from Biobank Japan (N=143,658)(56).

Biobank Japan collected baseline clinical information regarding kidney health through review of medical records. Serum creatinine measurements (using enzymatic essay) were retrieved from routine laboratory examination results in the medical records of participants. eGFR was estimated using the Japanese coefficient-modified CKD-EPI equation. CKD was defined as eGFR < 60 ml/min/1.73m^2^. Genotyping was undertaken using the Illumina HumanOmniExpressExome BeadChip or a combination of the Illumina HumanOmniExpress and HumanExome BeadChips. Genotypes were imputed with 1000 Genomes Project Phase 1 East Asian reference haplotypes (57). To reduce the variability in effect estimates and the bias caused by population stratification, the genetic associations were adjusted for age, sex, the top ten principal components of genetic ancestry, and any necessary trait-specific covariates (37).

The J-Kidney-Biobank/ToMMo cohort consisted of two datasets: the J-Kidney-Biobank and the Tohoku Medical Megabank Organization (ToMMo) study (58)(59). Subjects in J-Kidney-Biobank were recruited from seven medical institutes including Kyoto University, Kanazawa University, Niigata University, Okayama University, Kyushu University, the University of Tokyo, and Kawasaki Medical School. ToMMo, the Tohoku Medical Megabank Organization, conducted a prospective cohort study named "the Tohoku Medical Megabank Project Community-Based Cohort Study". The project was launched as part of the reconstruction following the Great East Japan Earthquake on 11 March 2011, with the aim of supporting personalized healthcare. Although the cohort study recruited residents in Miyagi or Iwate Prefecture, we only included the participants living in Miyagi Prefecture in the present study due to the genetic structure among participants living in Iwate Prefecture. In both datasets, CKD was defined as eGFR < 60mL/min/1.73m^2^ and/or the presence of urine abnormality. After selection, 382 CKD cases and 3,471 controls in the J-Kidney-Biobank (CKD cases recruited at baseline) as well as 3,664 cases and 5,952 controls in the ToMMo study (CKD cases identified in follow-up events) were included in the analysis. The samples were genotyped using the Japonica Array v2, and the genotypes were imputed with 3.5kJPNv2 reference panel of haplotypes (60). The GWAS of each dataset was conducted using BOLT-LMM (50), adjusted for sex, age and the first 10 principal components. A fixed effect meta-analysis of the two studies was conducted using METAL (61).

The China Kadoorie Biobank is a prospective cohort study of 500,000 Chinese adults (62). The baseline surveys for the study were conducted from 2004 to 2008 in 10 regions across China. All participants were followed-up for cause-specific mortality and morbidity through linkages with death certificates, disease surveillance systems, and health insurance databases. CKB has genotyped ~100K participants using custom designed Affymetrix Axiom arrays. CKD cases were defined as participants with a recorded ICD 10 code N18, and controls were defined as participants with no record of ICD 10 codes N00-N29 (other kidney conditions). After quality control, imputation was performed on 100,640 participants based on haplotypes derived from the 1000 Genomes Phase III. A GWAS was conducted for CKD (binary variable, N-cases= 461, N-controls= 94,887, 6.7% diabetes patients, 23.6% CKD cases with diabetes) using SAIGE (63), with adjustment for sex, age, region, genotyping array, and the first 12 principal components.

## Note S3. The three core assumptions of MR

Assumption 1: The genetic variant must be strongly associated with the exposure (NB the SNP need not to be the functional variant responsible for the SNP-exposure association). Typically, SNPs that pass genome-wide significance (P < 5 × 10^−8^) and have been replicated in an independent sample are used as instruments in MR studies. The use of weak instruments can bias MR estimates towards the observational estimate in one-sample MR settings and towards the null in two-sample MR settings (with non-overlapping samples). As common genetic variants frequently explain a small proportion of a trait’s variance, it is useful to consider the effects of multiple SNPs together. The causal effects of these SNPs on the outcome can then be combined using random-effect meta-analysis.

Assumption 2: The genetic variant should not be associated with confounders of the exposure-outcome relationship. Although this assumption cannot be tested, we can assess its plausibility by examining the association between the variant and known confounders of the exposure-outcome relationship.

Assumption 3: The genetic variant should only be related to the outcome of interest through the exposure of interest. This is commonly referred to as the “no pleiotropy” assumption or the exclusion restriction criterion. Horizontal pleiotropy, where a SNP is associated with multiple traits independently of the exposure of interest, potentially violates this assumption. While it is not possible to prove that this assumption holds in an MR study, various extensions of the basic MR design can be used to explore its presence and estimate the causal effect of the exposure mitigating the potential impact assumption violation.

## Note S4. Assessment of MR assumptions plausibility

We assessed the plausibility of the core Mendelian randomization assumptions using the following sensitivity analyses.

#### Assessing scope for pleiotropy

To test the third assumption (a lack of pleiotropic effects of the SNPs on the outcome, independent of the exposure), we used MR-Egger regression (64). The MR-Egger regression intercept was recorded as an indicator of pleiotropy. We also performed MR analyses using a weighted median MR (65) and mode estimate MR (66), which provides consistent causal estimates of the exposure on the outcome even when up to 50% of the information contributing to the analysis comes from genetic variants that exhibit pleiotropy. If all approaches (i.e. IVW MR, MR-Egger regression, weighted median MR and mode estimate MR) provide similar estimates, we can be more confident that our findings are robust.

#### Multivariable MR for correlated risk factors

We applied a multivariable inverse variance weighted method to identify the direct effects from correlated exposures (67)(68). Multivariable MR has an advantage over traditional MR in that it accounts for the potential pleiotropic influence of other exposures included in the analysis. Given the complex relationships between BMI, T2D and hypertension, we applied the multivariable inverse variance weighted method to the following three models to identify the independent causal effects of each of them on CKD:

1. CKD ~ BMI + T2D: Given the association between BMI and T2D (69), we conducted a multivariable MR to estimate the effect of each risk factor, independent of the other, on CKD incidence. We selected 701 SNPs associated with either BMI or T2D (or both) in Europeans as instruments for this analysis (**Table S25A**).
2. CKD ~ BMI + hypertension: Given the association between BMI and hypertension (70), we also conducted multivariable MR of BMI and hypertension on CKD (**Table S25B**);
3. CKD ~ BMI + T2D + hypertension: To take into account the complex relationship between T2D, BMI and hypertension, we conducted multivariable MR of BMI, T2D and hypertension on CKD (**Table S25C**);

These multivariable MR models provided strong evidence to support independent effects of BMI, genetic liability for hypertension and genetic liability for T2D on CKD. When all three factors were considered in the same multivariable MR model, BMI showed a strong association with CKD in CKDGen and UK Biobank. However, the evidence for an association between the genetic liability for hypertension with CKD and the genetic liability for T2D with CKD did not replicate across studies (**Table S15**).

We also performed an analysis that adjusted for apolipoprotein A-I and HDL cholesterol to assess the potential causal roles of HDL-related phenotypes in the development of CKD. In the setting of multivariable MR, we included all GWAS-associated SNPs for both apolipoprotein A-I and HDL cholesterol in this model. The genetic instruments of the multivariable MR model can be found in **Table S25D**.

#### Outlier identification

We conducted Radial MR (42) in the two sample analyses to identify outliers with the most weight in the MR analysis. Radial MR analysis was performed using modified second order weights and an α level of 0.05 divided by the number of SNPs being used to instrument the exposure.

#### Heterogeneity test

We conducted heterogeneity tests to estimate the variability in the causal estimates obtained for each SNP (i.e. how consistent is the causal estimate across all SNPs used as separate instruments). The Cochran’s Q test statistic was calculated to identify the presence of heterogeneity for the IVW MR (which is expected to be chi-squared distributed with degrees of freedom equal to the number of SNPs minus one) and Rucker’s Q’ statistic for MR Egger analyses (43). Lower heterogeneity suggests that violation of the MR assumptions – for instance, due to horizontal pleiotropy – is less likely (44).

#### Directionality test

To test the potential causal effect of CKD (and eGFR) on the CKD associated clinical risk factors, we took summary results data for CKD and eGFR as instrumental variables and conducted bidirectional MR (45). SNPs with a marginal P value smaller than 5×10^-8^ for CKD and eGFR were selected. Linkage disequilibrium (LD) clumping was conducted for the CKD and eGFR instruments with r^2^ threshold of 0.001. After LD clumping, 4 conditionally independent genetic variants were associated with CKD (55), 47 independent SNPs were associated with eGFR in Europeans (55) and 78 independent SNPs were associated with eGFR in East Asians (37) (instruments listed in **Table S26**). We found association information for these variants on BMI (13), HDL-c (16), hypertension (https://doi.org/10.5523/bris.2fahpksont1zi26xosyamqo8rr) and nephrolithiasis (14) in Europeans and BMI in East Asians (42). We performed IVW MR, weighted median MR and MR-Egger regression methods as described above.

## Note S5. Instrument strength estimation and power calculation

To quantify instrument strength, we calculated mean F-statistics for the 45 risk factors in Europeans (**Table S2**) and the 17 risk factors in East Asians (**Table S3**). F-statistic estimation has been described previously by Bowden et al (65). For the 17 risk factors with suitable instruments in both Europeans and East Asians, we compared the instrument strength for the same risk factors across the two ancestries (**Table S6**). In addition, power calculations were performed for each risk factor against CKD using the approximation that the sample size for an instrumental variable analysis is equal to that for the same regression analysis divided by the variance explained (r^2^) for the genetic variant on exposure (75). These calculations were performed using an online tool (<https://sb452.shinyapps.io/power/>) (76). The power of each risk factor on CKD can be found in Supplementary Table 1.

## Note S6. Non-linear MR

For non-linear MR analysis, we selected 73 BMI associated SNPs from the GIANT consortium (**Table S27**). We then estimated a weighted allele score for each participant by multiplying the number of BMI-increasing alleles the participant carried by the variant’s association with BMI from GIANT. For fasting glucose, we built the weighted allele score using 43 SNPs associated with fasting glucose from the MAGIC consortium (**Table S27**). In the UK Biobank and HUNT studies, we divided the sample into 100 strata using the residuals from a regression of the risk factor of interest (i.e. either BMI or fasting glucose) with adjustment for sex, age and genotyping array. For each stratum, we estimated the linear MR estimate as a localized average causal effect. We then performed meta-regression of the localized average causal effect estimates against the mean of the exposure (BMI and glucose) in each stratum in a flexible semiparametric framework by using the derivative of fractional polynomial models of degrees 2. Two tests for non-linearity are reported: a trend test, which assesses the linear trend among the localized average causal effect estimates, and a fractional polynomial test, which assesses whether a non-linear model fits the localized average causal effect estimates better than a linear model.

## Note S7. Additional limitations of the study

#### MR related limitations

MR is a helpful method to minimize several biases in observational studies, but the possibility of residual pleiotropy could bias estimates in this study. The likelihood of this bias is reduced because we observed consistent results across multiple MR estimators, which have different assumptions and different sources of bias. This increases our confidence that the estimates are not substantially biased by horizontal pleiotropy. Also, the MR-Egger regression test showed no clear directional pleiotropy for most of the risk factors. Furthermore, we were unable to account for the sample overlap between the exposure and outcome GWAS datasets. However, we used powerful instruments to estimate the relationship between the risk factors and the outcomes. Therefore, any sample overlap should not lead to substantial bias in our findings.

#### Other limitations related to ethnicities and CKD definition

As most of the study population was of European ancestry (46), our results should not be directly generalized to other ethnicities. Similarly, we could only assess 17 of the 45 clinical risk factors in East Asians. Further efforts are needed to obtain non-European samples and overcome the differences in data availability across populations.

For the phenotypes reflecting renal functions, glomerular filtration rate (GFR) was not directly measured but was estimated by an equation (CKD-EPI) (49) using serum creatinine, which may not reflect the actual GFR. For example, lower muscle mass will lead to lower creatinine, and further lead to higher eGFR. Despite this, the CKD-EPI equation is widely used and has been applied in most of the recent genetic studies of renal function (54)(37).

## References

1. Elsworth B, Dawe K, Vincent EE, Langdon R, Lynch BM, Martin RM, et al. MELODI: Mining Enriched Literature Objects to Derive Intermediates. Int J Epidemiol. 2018 Jan 12.

2. Elsworth B, Gaunt TR. MELODI Presto: A fast and agile tool to explore semantic triples derived from biomedical literature. Bioinformatics. 2020 Aug 18.

3. Hemani G, Zheng J, Elsworth B, Wade KH, Haberland V, Baird D, et al. The MR-Base platform supports systematic causal inference across the human phenome. Elife. 2018 May 30;7.

4. Evangelou E, Warren HR, Mosen-Ansorena D, Mifsud B, Pazoki R, Gao H, et al. Genetic analysis of over 1 million people identifies 535 new loci associated with blood pressure traits. Nat Genet. 2018 Oct;50(10):1412–25.

5. Mahajan A, Taliun D, Thurner M, Robertson NR, Torres JM, Rayner NW, et al. Fine-mapping type 2 diabetes loci to single-variant resolution using high-density imputation and islet-specific epigenome maps. Nat Genet. 2018 Nov;50(11):1505–13.

6. Soranzo N, Sanna S, Wheeler E, Gieger C, Radke D, Dupuis J, et al. Common variants at 10 genomic loci influence hemoglobin A₁(C) levels via glycemic and nonglycemic pathways. Diabetes. 2010 Dec;59(12):3229–39.

7. Scott RA, Lagou V, Welch RP, Wheeler E, Montasser ME, Luan J, et al. Large-scale association analyses identify new loci influencing glycemic traits and provide insight into the underlying biological pathways. Nat Genet. 2012 Sep;44(9):991–1005.

8. Wootton RE, Richmond RC, Stuijfzand BG, Lawn RB, Sallis HM, Taylor GMJ, et al. Causal effects of lifetime smoking on risk for depression and schizophrenia: Evidence from a Mendelian randomisation study. BioRxiv 2018. https://www.biorxiv.org/content/10.1101/381301v1

9. Warrington NM, Beaumont RN, Horikoshi M, Day FR, Helgeland Ø, Laurin C, et al. Maternal and fetal genetic effects on birth weight and their relevance to cardio-metabolic risk factors. Nat Genet. 2019 May;51(5):804–14.

10. Jones SE, Lane JM, Wood AR, van Hees VT, Tyrrell J, Beaumont RN, et al. Genome-wide association analyses of chronotype in 697,828 individuals provides insights into circadian rhythms. Nat Commun. 2019 Jan 29;10(1):343.

11. Dashti HS, Jones SE, Wood AR, Lane JM, van Hees VT, Wang H, et al. Genome-wide association study identifies genetic loci for self-reported habitual sleep duration supported by accelerometer-derived estimates. Nat Commun. 2019 Mar 7;10(1):1100.

12. Lane JM, Jones SE, Dashti HS, Wood AR, Aragam KG, van Hees VT, et al. Biological and clinical insights from genetics of insomnia symptoms. Nat Genet. 2019 Mar;51(3):387–93.

13. Yengo L, Sidorenko J, Kemper KE, Zheng Z, Wood AR, Weedon MN, et al. Meta-analysis of genome-wide association studies for height and body mass index in ~700,000 individuals of European ancestry. BioRxiv 2018. https://www.biorxiv.org/content/early/2018/03/02/274654

14. Oddsson A, Sulem P, Helgason H, Edvardsson VO, Thorleifsson G, Sveinbjörnsson G, et al. Common and rare variants associated with kidney stones and biochemical traits. Nat Commun. 2015 Aug 14;6:7975.

15. Sulem P, Gudbjartsson DF, Walters GB, Helgadottir HT, Helgason A, Gudjonsson SA, et al. Identification of low-frequency variants associated with gout and serum uric acid levels. Nat Genet. 2011 Oct 9;43(11):1127–30.

16. Liu DJ, Peloso GM, Yu H, Butterworth AS, Wang X, Mahajan A, et al. Exome-wide association study of plasma lipids in >300,000 individuals. Nat Genet. 2017 Dec;49(12):1758–66.

17. Clarke T-K, Adams MJ, Davies G, Howard DM, Hall LS, Padmanabhan S, et al. Genome-wide association study of alcohol consumption and genetic overlap with other health-related traits in UK Biobank (N=112 117). Mol Psychiatry. 2017 Oct;22(10):1376–84.

18. Dupuis J, Langenberg C, Prokopenko I, Saxena R, Soranzo N, Jackson AU, et al. New genetic loci implicated in fasting glucose homeostasis and their impact on type 2 diabetes risk. Nat Genet. 2010 Feb;42(2):105–16.

19. Teumer A, Qi Q, Nethander M, Aschard H, Bandinelli S, Beekman M, et al. Genomewide meta-analysis identifies loci associated with IGF-I and IGFBP-3 levels with impact on age-related traits. Aging Cell. 2016 Oct;15(5):811–24.

20. Kilpeläinen TO, Carli JFM, Skowronski AA, Sun Q, Kriebel J, Feitosa MF, et al. Genome-wide meta-analysis uncovers novel loci influencing circulating leptin levels. Nat Commun. 2016 Feb 1;7:10494.

21. Dastani Z, Hivert M-F, Timpson N, Perry JRB, Yuan X, Scott RA, et al. Novel loci for adiponectin levels and their influence on type 2 diabetes and metabolic traits: a multi-ethnic meta-analysis of 45,891 individuals. PLoS Genet. 2012 Mar 29;8(3):e1002607.

22. Richardson TG, Sanderson E, Palmer TM, Ala-Korpela M, Ference BA, Davey Smith G, et al. Evaluating the relationship between circulating lipoprotein lipids and apolipoproteins with risk of coronary heart disease: A multivariable Mendelian randomisation analysis. PLoS Med. 2020 Mar;17(3):e1003062.

23. Nikpay M, Goel A, Won H-H, Hall LM, Willenborg C, Kanoni S, et al. A comprehensive 1,000 Genomes-based genome-wide association meta-analysis of coronary artery disease. Nat Genet. 2015 Oct;47(10):1121–30.

24. Morris JA, Kemp JP, Youlten SE, Laurent L, Logan JG, Chai RC, et al. An atlas of genetic influences on osteoporosis in humans and mice. Nat Genet. 2019 Feb;51(2):258–66.

25. van Meurs JBJ, Pare G, Schwartz SM, Hazra A, Tanaka T, Vermeulen SH, et al. Common genetic loci influencing plasma homocysteine concentrations and their effect on risk of coronary artery disease. Am J Clin Nutr. 2013 Sep;98(3):668–76.

26. Han X, Ong J-S, An J, Hewitt AW, Gharahkhani P, MacGregor S. Using Mendelian randomization to evaluate the causal relationship between serum C-reactive protein levels and age-related macular degeneration. Eur J Epidemiol. 2020 Feb;35(2):139–46.

27. Meyer TE, Verwoert GC, Hwang S-J, Glazer NL, Smith AV, van Rooij FJA, et al. Genome-wide association studies of serum magnesium, potassium, and sodium concentrations identify six Loci influencing serum magnesium levels. PLoS Genet. 2010 Aug 5;6(8). http://dx.doi.org/10.1371/journal.pgen.1001045

28. Pazoki R, Evangelou E, Mosen-Ansorena D, Pinto RC, Karaman I, Blakeley P, et al. GWAS for urinary sodium and potassium excretion highlights pathways shared with cardiovascular traits. Nat Commun. 2019 Aug 13;10(1):3653.

29. Kestenbaum B, Glazer NL, Köttgen A, Felix JF, Hwang S-J, Liu Y, et al. Common genetic variants associate with serum phosphorus concentration. J Am Soc Nephrol. 2010 Jul;21(7):1223–32.

30. Benyamin B, Esko T, Ried JS, Radhakrishnan A, Vermeulen SH, Traglia M, et al. Novel loci affecting iron homeostasis and their effects in individuals at risk for hemochromatosis. Nat Commun. 2014 Oct 29;5:4926.

31. Evans DM, Zhu G, Dy V, Heath AC, Madden PAF, Kemp JP, et al. Genome-wide association study identifies loci affecting blood copper, selenium and zinc. Hum Mol Genet. 2013 Oct 1;22(19):3998–4006.

32. Ferrucci L, Perry JRB, Matteini A, Perola M, Tanaka T, Silander K, et al. Common variation in the beta-carotene 15,15’-monooxygenase 1 gene affects circulating levels of carotenoids: a genome-wide association study. Am J Hum Genet. 2009 Feb;84(2):123–33.

33. Mondul AM, Yu K, Wheeler W, Zhang H, Weinstein SJ, Major JM, et al. Genome-wide association study of circulating retinol levels. Hum Mol Genet. 2011 Dec 1;20(23):4724–31.

34. Jiang X, O’Reilly PF, Aschard H, Hsu Y-H, Richards JB, Dupuis J, et al. Genome-wide association study in 79,366 European-ancestry individuals informs the genetic architecture of 25-hydroxyvitamin D levels. Nat Commun. 2018 Jan 17;9(1):260.

35. Tanaka T, Scheet P, Giusti B, Bandinelli S, Piras MG, Usala G, et al. Genome-wide association study of vitamin B6, vitamin B12, folate, and homocysteine blood concentrations. Am J Hum Genet. 2009 Apr;84(4):477–82.

36. Teumer A, Chaker L, Groeneweg S, Li Y, Di Munno C, Barbieri C, et al. Genome-wide analyses identify a role for SLC17A4 and AADAT in thyroid hormone regulation. Nat Commun. 2018 Oct 26;9(1):4455.

37. Kanai M, Akiyama M, Takahashi A, Matoba N, Momozawa Y, Ikeda M, et al. Genetic analysis of quantitative traits in the Japanese population links cell types to complex human diseases. Nat Genet. 2018 Mar;50(3):390–400.

38. Takeuchi F, Akiyama M, Matoba N, Katsuya T, Nakatochi M, Tabara Y, et al. Interethnic analyses of blood pressure loci in populations of East Asian and European descent. Nat Commun. 2018 Nov 28;9(1):5052.

39. Cho YS, Chen C-H, Hu C, Long J, Ong RTH, Sim X, et al. Meta-analysis of genome-wide association studies identifies eight new loci for type 2 diabetes in east Asians. Nat Genet. 2011 Dec 11;44(1):67–72.

40. Matoba N, Akiyama M, Ishigaki K, Kanai M, Takahashi A, Momozawa Y, et al. GWAS of smoking behaviour in 165,436 Japanese people reveals seven new loci and shared genetic architecture. Nat Hum Behav. 2019 May;3(5):471–7.

41. Nishiyama T, Nakatochi M, Goto A, Iwasaki M, Hachiya T, Sutoh Y, et al. Genome-wide association meta-analysis and Mendelian randomization analysis confirm the influence of ALDH2 on sleep durationin the Japanese population. Sleep. 2019 Jun 11;42(6).

42. Akiyama M, Okada Y, Kanai M, Takahashi A, Momozawa Y, Ikeda M, et al. Genome-wide association study identifies 112 new loci for body mass index in the Japanese population. Nat Genet. 2017 Oct;49(10):1458–67.

43. Urabe Y, Tanikawa C, Takahashi A, Okada Y, Morizono T, Tsunoda T, et al. A genome-wide association study of nephrolithiasis in the Japanese population identifies novel susceptible Loci at 5q35.3, 7p14.3, and 13q14.1. PLoS Genet. 2012 Mar 1;8(3):e1002541.

44. Jorgenson E, Thai KK, Hoffmann TJ, Sakoda LC, Kvale MN, Banda Y, et al. Genetic contributors to variation in alcohol consumption vary by race/ethnicity in a large multi-ethnic genome-wide association study. Mol Psychiatry. 2017 Sep;22(9):1359–67.

45. Millwood IY, Walters RG, Mei XW, Guo Y, Yang L, Bian Z, et al. Conventional and genetic evidence on alcohol and vascular disease aetiology: a prospective study of 500 000 men and women in China. Lancet. 2019 May 4;393(10183):1831–42.

46. Lu X, Wang L, Chen S, He L, Yang X, Shi Y, et al. Genome-wide association study in Han Chinese identifies four new susceptibility loci for coronary artery disease. Nat Genet. 2012 Jul 1;44(8):890–4.

47. Okada Y, Takahashi A, Ohmiya H, Kumasaka N, Kamatani Y, Hosono N, et al. Genome-wide association study for C-reactive protein levels identified pleiotropic associations in the IL6 locus. Hum Mol Genet. 2011 Mar 15;20(6):1224–31.

48. Spracklen CN, Horikoshi M, Kim YJ, Lin K, Bragg F, Moon S, et al. Identification of type 2 diabetes loci in 433,540 East Asian individuals. Nature. 2020 May 6.

49. Levey AS, Stevens LA, Schmid CH, Zhang YL, Castro AF 3rd, Feldman HI, et al. A new equation to estimate glomerular filtration rate. Ann Intern Med. 2009 May 5;150(9):604–12.

50. Loh P-R, Tucker G, Bulik-Sullivan BK, Vilhjálmsson BJ, Finucane HK, Salem RM, et al. Efficient Bayesian mixed-model analysis increases association power in large cohorts. Nat Genet. 2015 Mar;47(3):284–90.

51. Krokstad S, Langhammer A, Hveem K, Holmen TL, Midthjell K, Stene TR, et al. Cohort Profile: the HUNT Study, Norway. Int J Epidemiol. 2013 Aug;42(4):968–77.

52. Das S, Forer L, Schönherr S, Sidore C, Locke AE, Kwong A, et al. Next-generation genotype imputation service and methods. Nat Genet. 2016 Oct;48(10):1284–7.

53. McCarthy S, Das S, Kretzschmar W, Delaneau O, Wood AR, Teumer A, et al. A reference panel of 64,976 haplotypes for genotype imputation. Nat Genet. 2016 Oct;48(10):1279–83.

54. Wuttke M, Li Y, Li M, Sieber KB, Feitosa MF, Gorski M, et al. A catalog of genetic loci associated with kidney function from analyses of a million individuals. Nat Genet. 2019 Jun;51(6):957–72.

55. Pattaro C, Teumer A, Gorski M, Chu AY, Li M, Mijatovic V, et al. Genetic associations at 53 loci highlight cell types and biological pathways relevant for kidney function. Nat Commun. 2016 Jan 21;7:10023.

56. Nagai A, Hirata M, Kamatani Y, Muto K, Matsuda K, Kiyohara Y, et al. Overview of the BioBank Japan Project: Study design and profile. J Epidemiol. 2017 Mar;27(3S):S2–8.

57. 1000 Genomes Project Consortium, Auton A, Brooks LD, Durbin RM, Garrison EP, Kang HM, et al. A global reference for human genetic variation. Nature. 2015 Oct 1;526(7571):68–74.

58. Kuriyama S, Yaegashi N, Nagami F, Arai T, Kawaguchi Y, Osumi N, et al. The Tohoku Medical Megabank Project: Design and Mission. J Epidemiol. 2016 Sep 5;26(9):493–511.

59. Fuse N, Sakurai-Yageta M, Katsuoka F, Danjoh I, Shimizu R, Tamiya G, et al. Establishment of Integrated Biobank for Precision Medicine and Personalized Healthcare: The Tohoku Medical Megabank Project. JMA Journal. 2019;2(2):113–22.

60. Tadaka S, Katsuoka F, Ueki M, Kojima K, Makino S, Saito S, et al. 3.5KJPNv2: an allele frequency panel of 3552 Japanese individuals including the X chromosome. Hum Genome Var. 2019 Jun 18;6:28.

61. Willer CJ, Li Y, Abecasis GR. METAL: fast and efficient meta-analysis of genomewide association scans. Bioinformatics. 2010 Sep 1;26(17):2190–1.

62. Chen Z, Chen J, Collins R, Guo Y, Peto R, Wu F, et al. China Kadoorie Biobank of 0.5 million people: survey methods, baseline characteristics and long-term follow-up. Int J Epidemiol. 2011 Dec;40(6):1652–66.

63. Zhou W, Nielsen JB, Fritsche LG, Dey R, Gabrielsen ME, Wolford BN, et al. Efficiently controlling for case-control imbalance and sample relatedness in large-scale genetic association studies. Nat Genet. 2018 Sep;50(9):1335–41.

64. Bowden J, Davey Smith G, Burgess S. Mendelian randomization with invalid instruments: effect estimation and bias detection through Egger regression. Int J Epidemiol. 2015 Apr;44(2):512–25.

65. Bowden J, Del Greco M F, Minelli C, Davey Smith G, Sheehan NA, Thompson JR. Assessing the suitability of summary data for two-sample Mendelian randomization analyses using MR-Egger regression: the role of the I 2 statistic. Int J Epidemiol. 2016;45(6):1961–74.

66. Hartwig FP, Davey Smith G, Bowden J. Robust inference in summary data Mendelian randomization via the zero modal pleiotropy assumption. Int J Epidemiol. 2017 Jul 12.

67. Sanderson E, Davey Smith G, Windmeijer F, Bowden J. An examination of multivariable Mendelian randomization in the single-sample and two-sample summary data settings. Int J Epidemiol. 2018 Dec 10.

68. Burgess S, Thompson SG. Multivariable Mendelian randomization: the use of pleiotropic genetic variants to estimate causal effects. Am J Epidemiol. 2015 Feb 15;181(4):251–60.

69. Corbin LJ, Richmond RC, Wade KH, Burgess S, Bowden J, Smith GD, et al. BMI as a Modifiable Risk Factor for Type 2 Diabetes: Refining and Understanding Causal Estimates Using Mendelian Randomization. Diabetes. 2016 Oct;65(10):3002–7.

70. Lee M-R, Lim Y-H, Hong Y-C. Causal association of body mass index with hypertension using a Mendelian randomization design. Medicine. 2018 Jul;97(30):e11252.

71. Bowden J, Spiller W, Del Greco M F, Sheehan N, Thompson J, Minelli C, et al. Improving the visualization, interpretation and analysis of two-sample summary data Mendelian randomization via the Radial plot and Radial regression. Int J Epidemiol. 2018 Aug 1;47(4):1264–78.

72. Bowden J, Del Greco M F, Minelli C, Zhao Q, Lawlor DA, Sheehan NA, et al. Improving the accuracy of two-sample summary-data Mendelian randomization: moving beyond the NOME assumption. Int J Epidemiol. 2018 Dec 18.

73. Zheng J, Baird D, Borges M-C, Bowden J, Hemani G, Haycock P, et al. Recent Developments in Mendelian Randomization Studies. Curr Epidemiol Rep. 2017 Nov 22;4(4):330–45.

74. Timpson NJ, Nordestgaard BG, Harbord RM, Zacho J, Frayling TM, Tybjærg-Hansen A, et al. C-reactive protein levels and body mass index: elucidating direction of causation through reciprocal Mendelian randomization. Int J Obes. 2011 Feb;35(2):300–8.

75. Freeman G, Cowling BJ, Schooling CM. Power and sample size calculations for Mendelian randomization studies using one genetic instrument. Int J Epidemiol. 2013 Aug;42(4):1157–63.

76. Burgess S. Sample size and power calculations in Mendelian randomization with a single instrumental variable and a binary outcome. Int J Epidemiol. 2014 Jun;43(3):922–9.

77. Mills MC, Rahal C. A scientometric review of genome-wide association studies. Commun Biol. 2019 Jan 7;2:9.

# Supplementary Figures


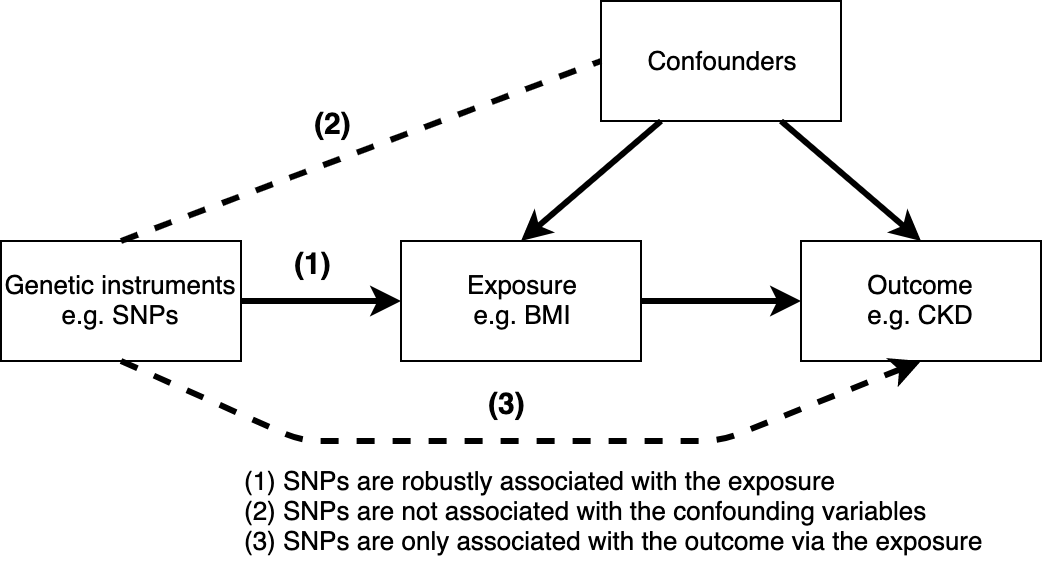


**Figure S1**. The Mendelian randomization analysis model and the three key assumptions.


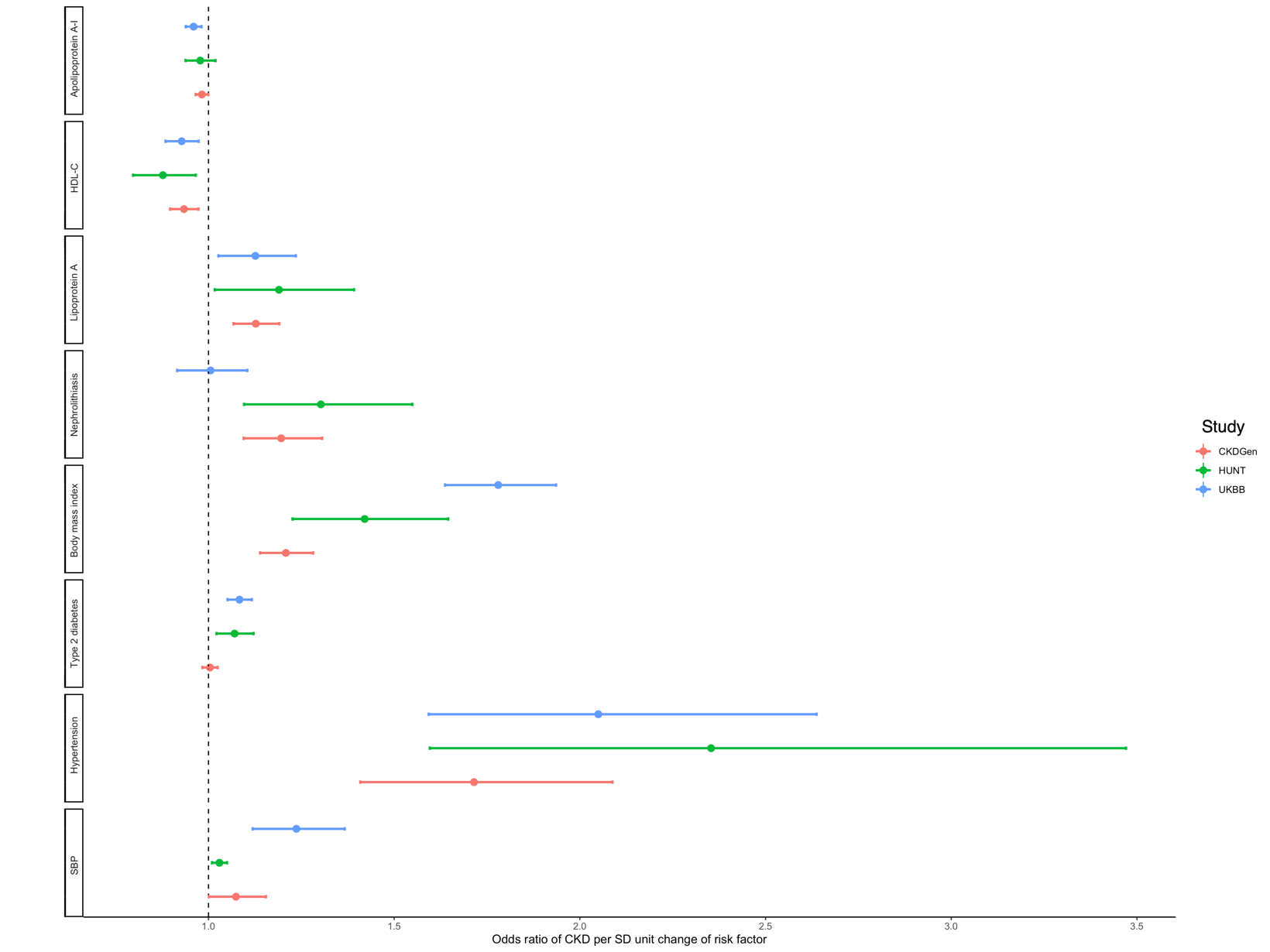

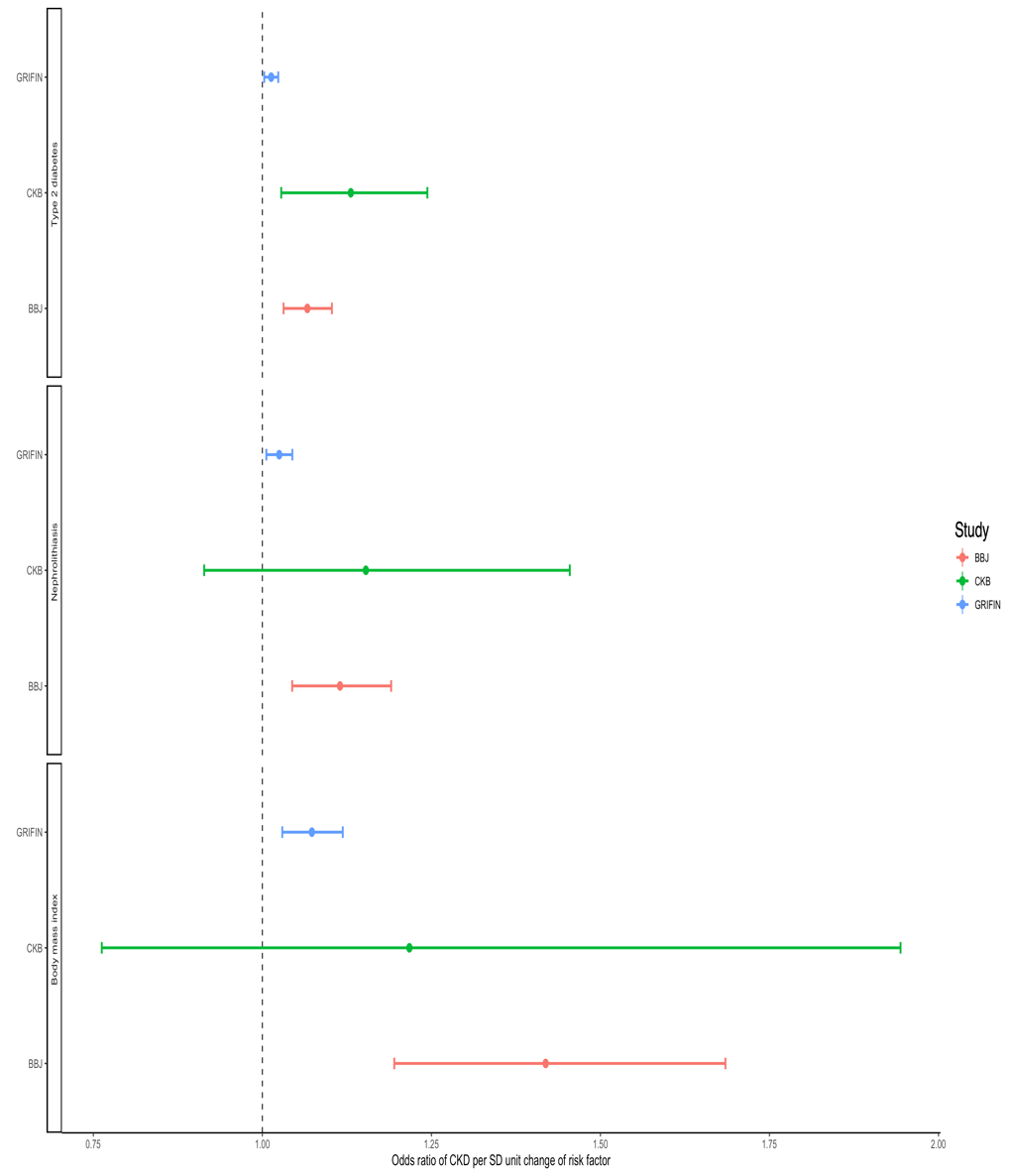


**Figure S2**. Forest plot for causal effects of the risk factors with reliable causal effects on CKD in European and Eastern Asians. A) this plot represents causal estimates of eight risk factors on CKD in Europeans; B) this plot represents causal estimates of three risk factors on CKD in East Asians.


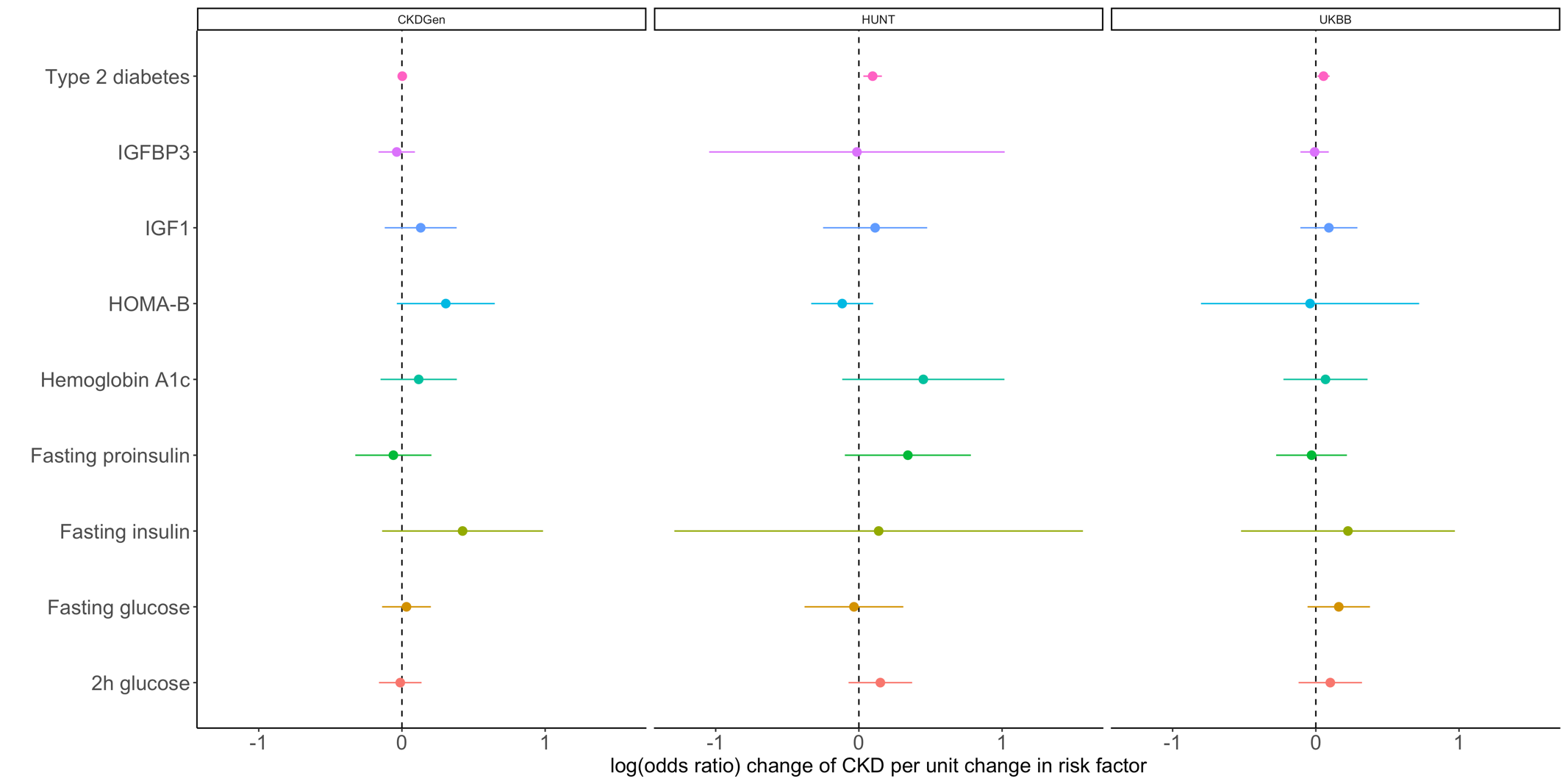


**Figure S3**. Forest plot for causal effects of T2D and eight glycemic phenotypes on CKD risk. The subplots represent MR results using outcome data from CKDGen consortium, HUNT Study, and UK Biobank, respectively.


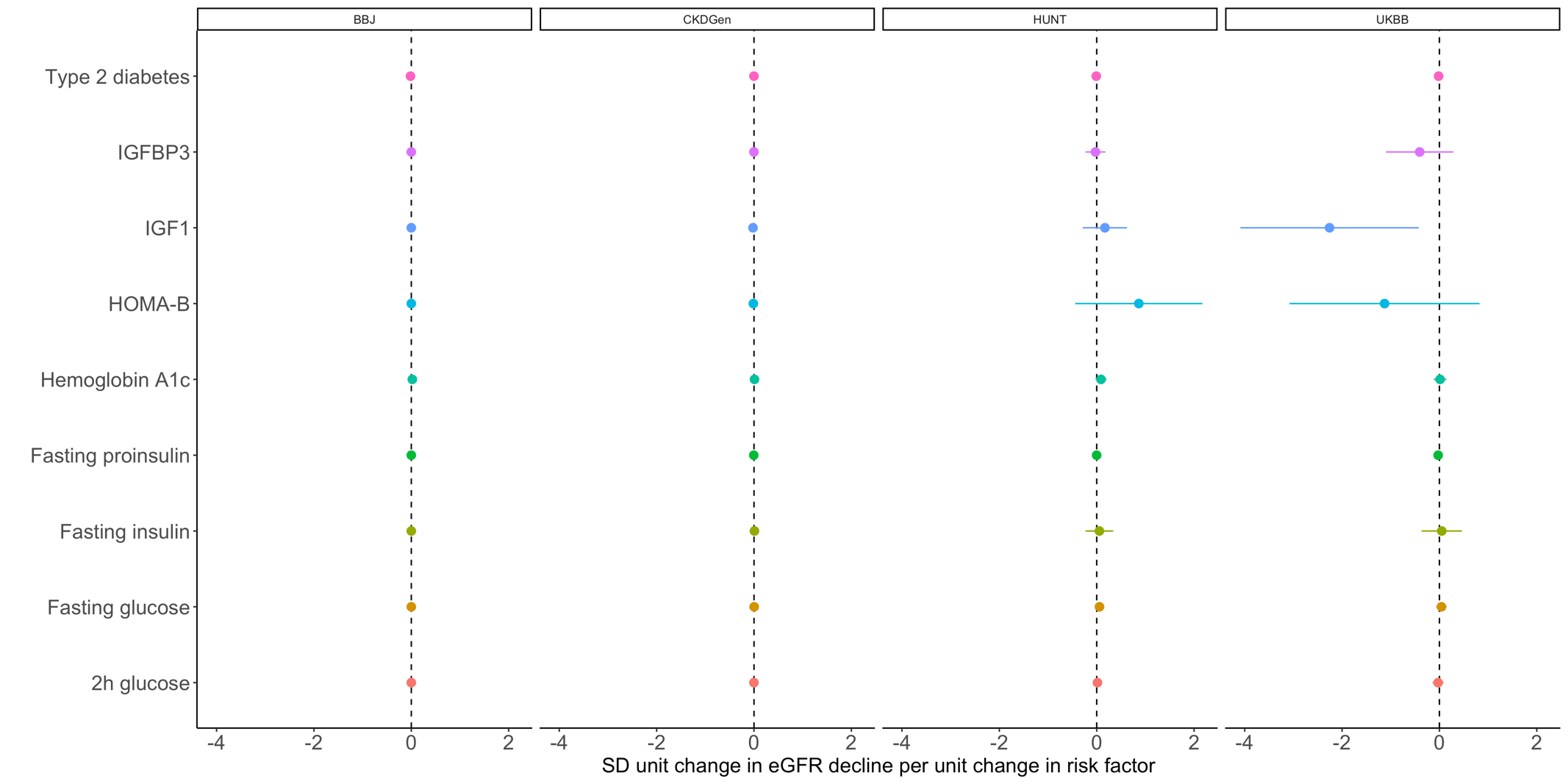


**Figure S4**. Forest plot for causal effects of T2D and eight glycemic phenotypes on eGFR. The subplots represent MR results using outcome data from Biobank Japan, CKDGen consortium, HUNT Study, and UK Biobank, respectively.


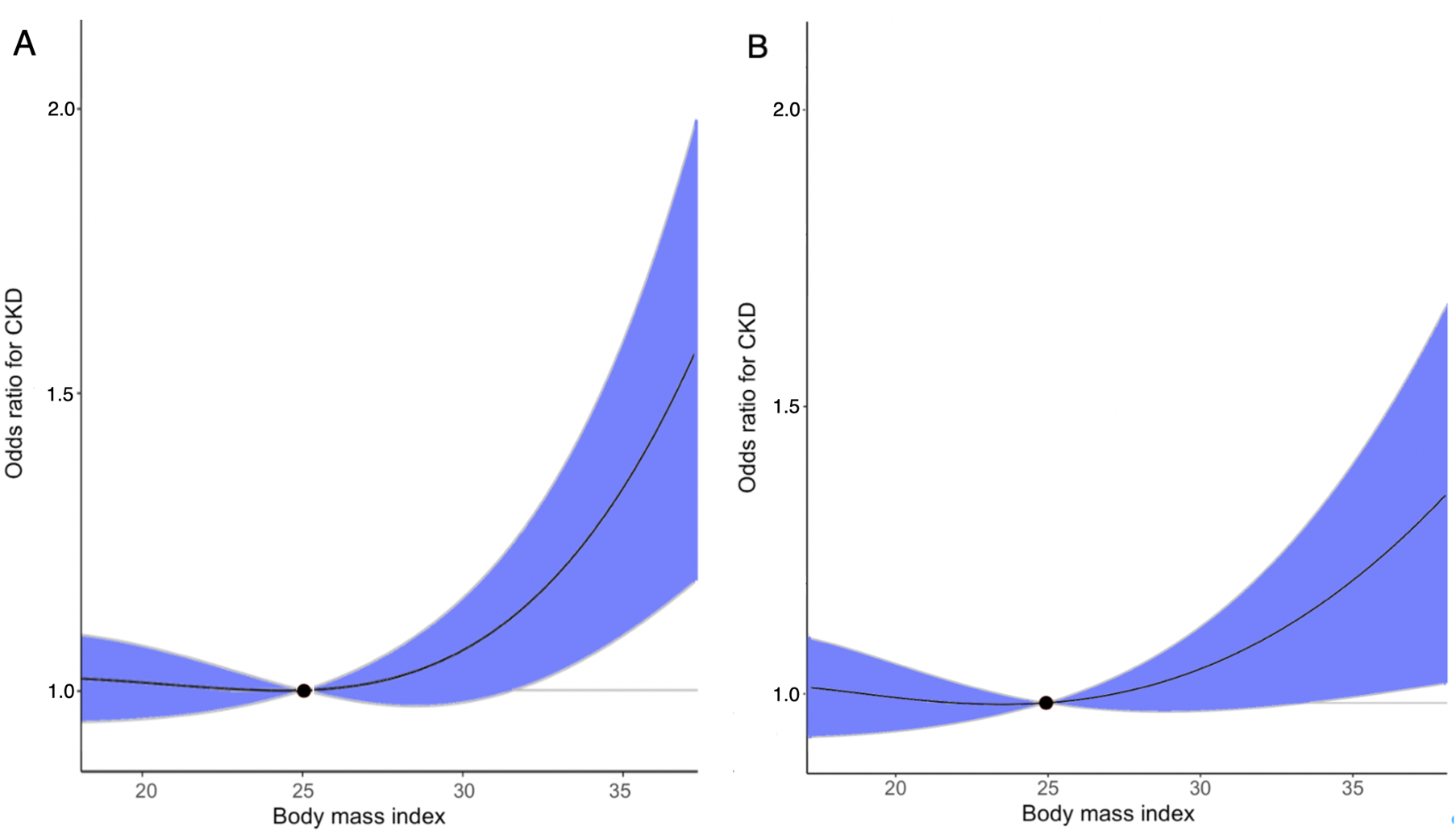


**Figure S5**. Non-linear Mendelian randomization of body mass index on chronic kidney disease risk in men and women for UK Biobank. Gradient at each point of the curve is the localized average causal effect. Shaded areas represent 95% confidence intervals. Plot A and B for male and female in UK Biobank.


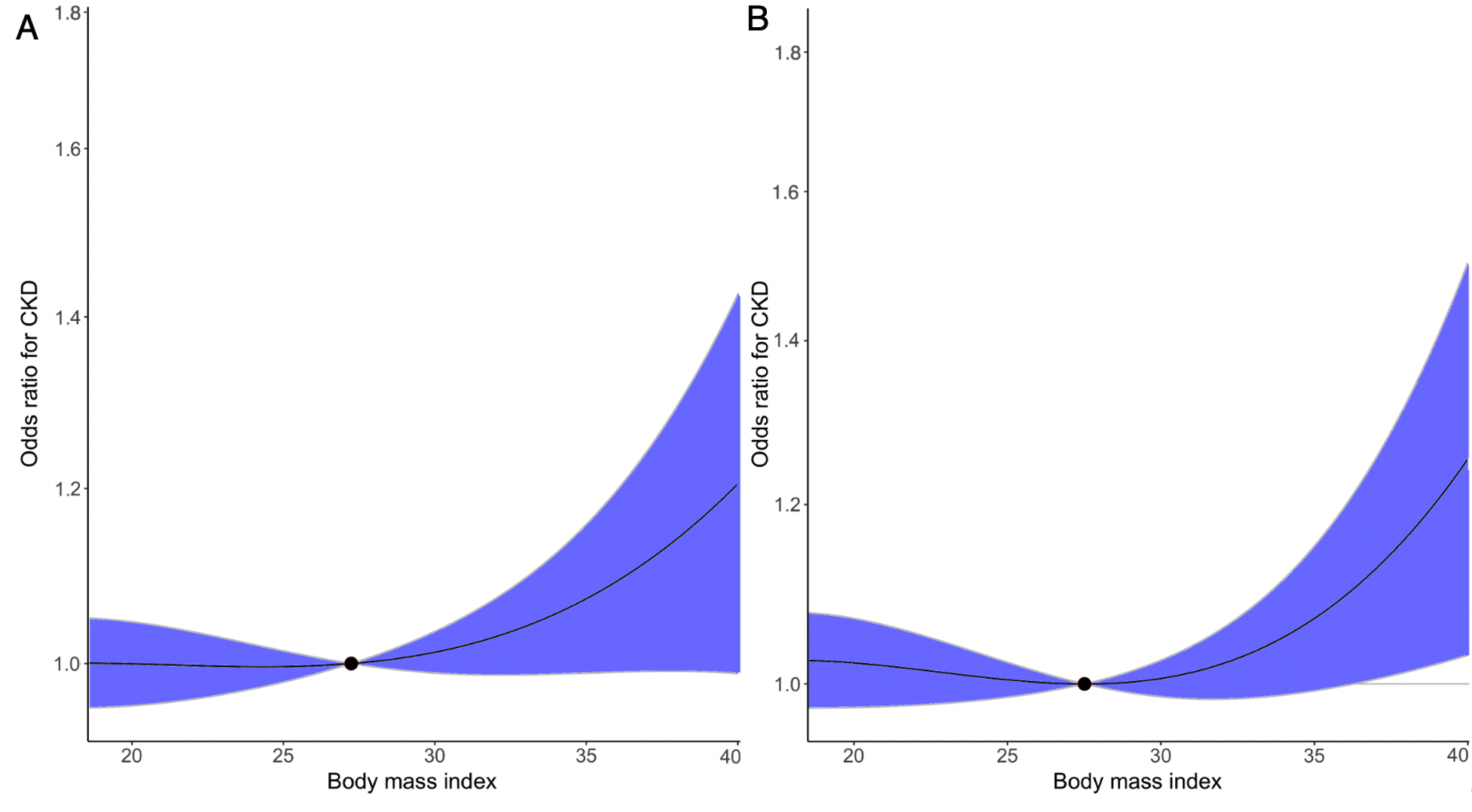


**Figure S6**. Non-linear Mendelian randomization of body mass index on chronic kidney disease risk in younger (<65) and elder participations (>=65) for UK Biobank. Gradient at each point of the curve is the localized average causal effect. Shaded areas represent 95% confidence intervals. Plot A and B for younger and elder participations in UK Biobank.


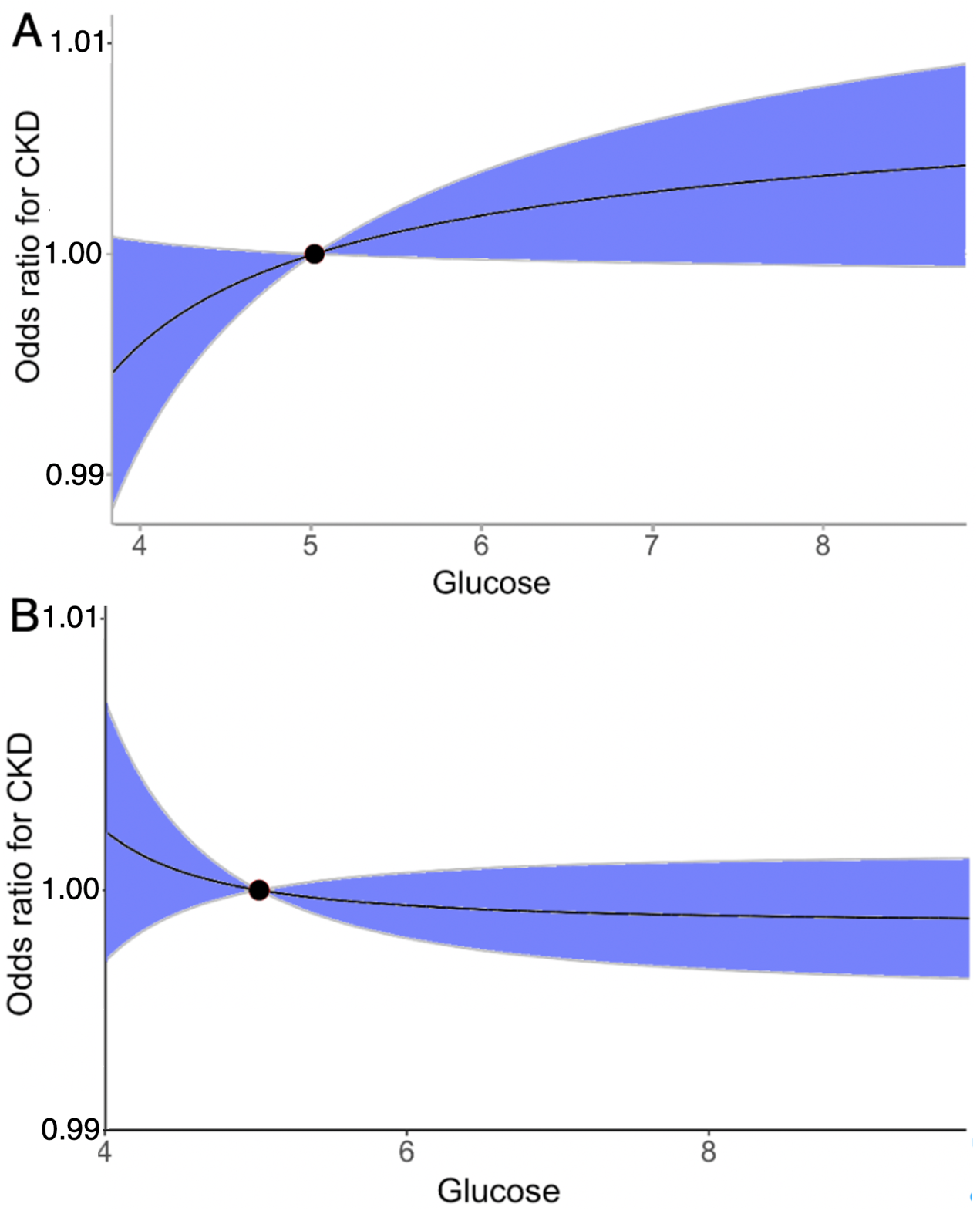


**Figure S7**. Non-linear Mendelian randomization of glucose on chronic kidney disease risk for UK Biobank (plot A) and HUNT Study (plot B). Gradient at each point of the curve is the localized average causal effect. Shaded areas represent 95% confidence intervals.


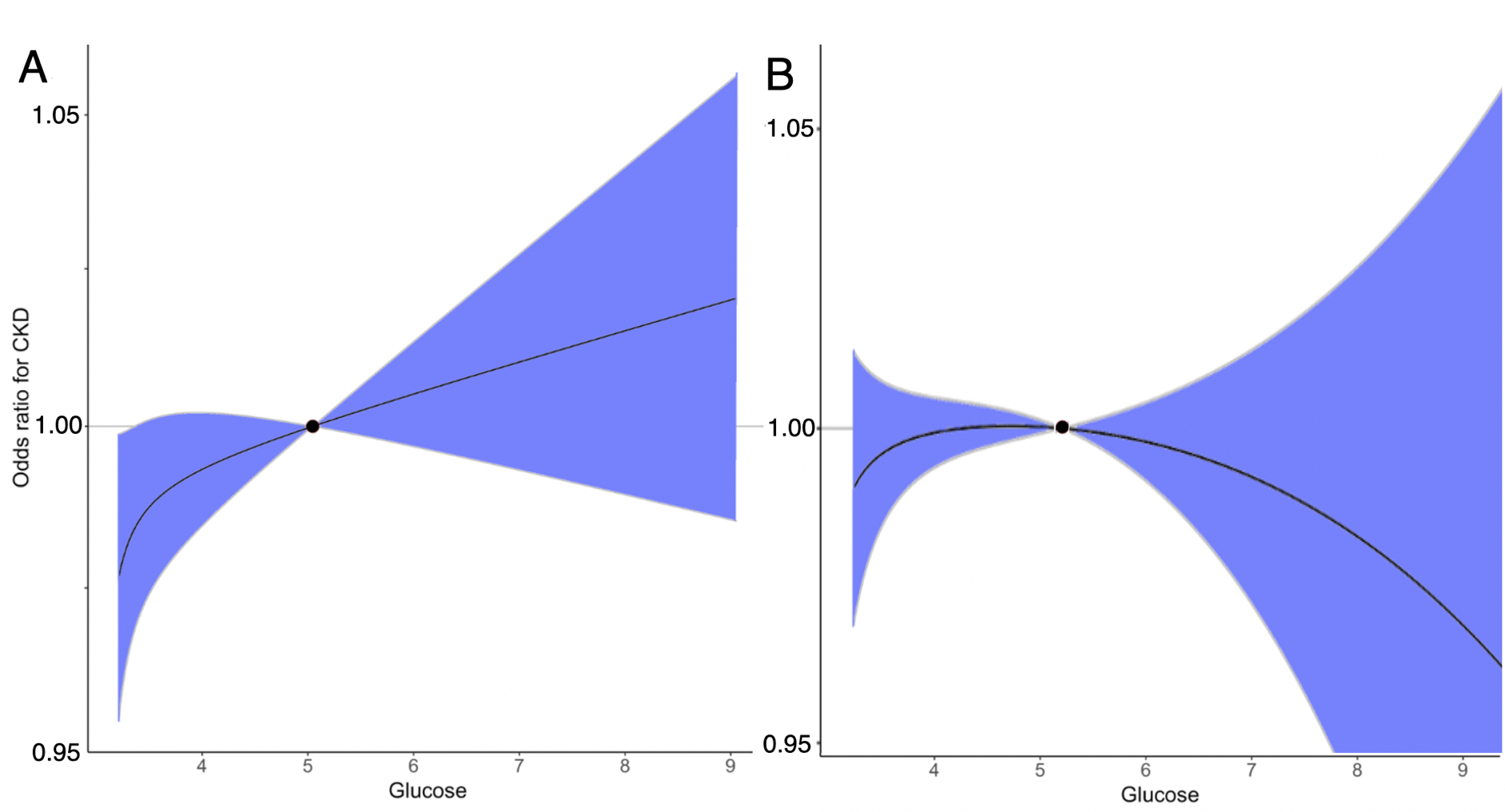


**Figure S8**. Non-linear Mendelian randomization of glucose on chronic kidney disease risk in men (plot A) and women (plot B) for UK Biobank. Gradient at each point of the curve is the localized average causal effect. Shaded areas represent 95% confidence intervals. Plot A and B for male and female in UK Biobank.


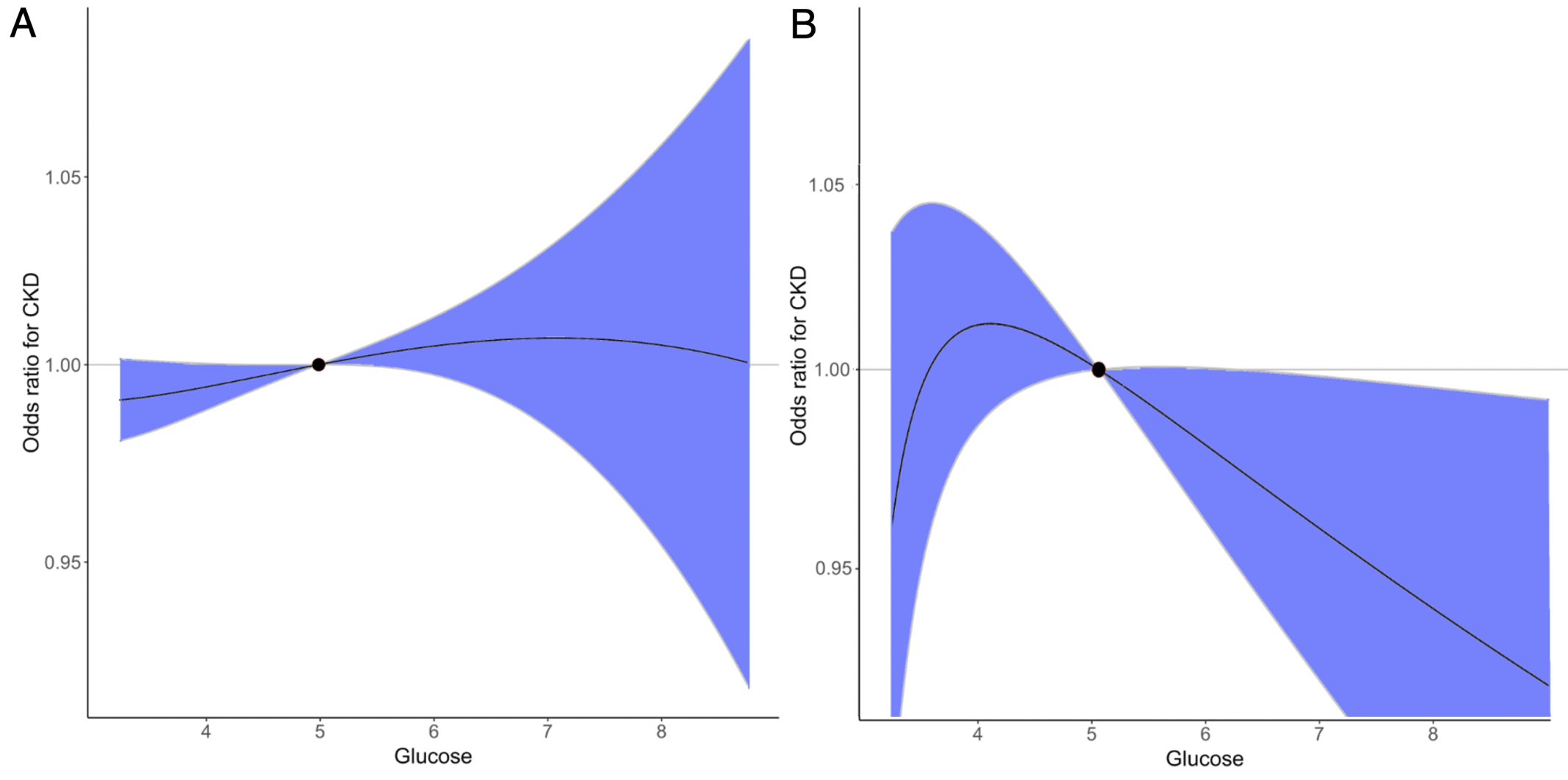


**Figure S9**. Non-linear Mendelian randomization of glucose on chronic kidney disease risk in younger (<65) and elder (>=65) participations for UK Biobank. Gradient at each point of the curve is the localized average causal effect. Shaded areas represent 95% confidence intervals. Plot A and B for younger and elder participations in UK Biobank.
